# Knowledge-Based Feature Selection Substantially Enhances Data-Driven Wastewater Treatment Modeling

**DOI:** 10.1021/acs.est.6c04963

**Published:** 2026-07-12

**Authors:** Senyuan Gu, Shuting Wang, Ruihong Qiu, Kaili Li, Jaswinder Manjeet Singh, Jue Zhang, Bing-Jie Ni, T. David Waite, Liu Ye, Haoran Duan

**Affiliations:** † UNSW Water Research Centre, School of Civil and Environmental Engineering, The University of New South Wales, Sydney, New South Wales 2052, Australia; ‡ Australian Centre for Water and Environmental Biotechnology (ACWEB, formerly AWMC), 1974 The University of Queensland, Brisbane, Queensland 4072, Australia; § School of Chemical Engineering, 1974 The University of Queensland, Brisbane, Queensland 4072, Australia; ∥ School of Information Technology and Electrical Engineering, 1974 The University of Queensland, Brisbane, Queensland 4072, Australia; ⊥ 124729 Gold Coast City Council, Southport, Queensland 4215, Australia; # College of Geo-informatics, Zhejiang University of Technology, Hangzhou, Zhejiang Province 310000, P. R. China; ∇ UNSW Centre for Transformational Environmental Technologies, Yixing, Jiangsu Province 214206, P. R. China; ○ Department of Civil Engineering, 25809 The University of Hong Kong, Pokfulam, Hong Kong SAR 999077, China

**Keywords:** Feature selection, wastewater modeling, high-dimensionality data, expert knowledge, machine learning, data driven

## Abstract

Data-driven modeling in wastewater treatment is increasingly constrained by the reality of small, high-dimensional data, where the abundant monitoring parameters in small-sized data sets obscure fundamental mechanistic understandings. This study proposes a knowledge-driven feature selection framework that integrates mechanistic insights with statistical correlations to identify the most informative predictive features. Using nitrous oxide (N_2_O) emission prediction at a full-scale plant as a case study, we compared classic deep-learning feature selection algorithms using attention mechanisms against two new knowledge-based approaches: (i) expert-guided feature selection and (ii) large language model (LLM)-augmented feature selection. Expert-knowledge-guided feature selection substantially enhances predictive accuracy, achieving a mean R^2^ of 0.723 and an MAE of 0.033, compared to R^2^ = 0.712 and MAE = 0.033 for the best-performing attention-based architecture. More importantly, the proposed framework markedly improves model generalizability: under out-of-distribution high-flow conditions where the attention-based model fails to capture N_2_O emission patterns, the expert-selected model continues to reproduce the dominant temporal dynamics of N_2_O emissions. The LLM-assisted approach also delivers competitive accuracy (mean R^2^ = 0.596, MAE = 0.041) and similarly preserves generalizability under an input distributional shift. By introducing mechanistic understanding into the feature selection process, this framework offers a generalizable pathway for addressing complex wastewater treatment challenges while maintaining a computational efficiency.

## Graphical abstract

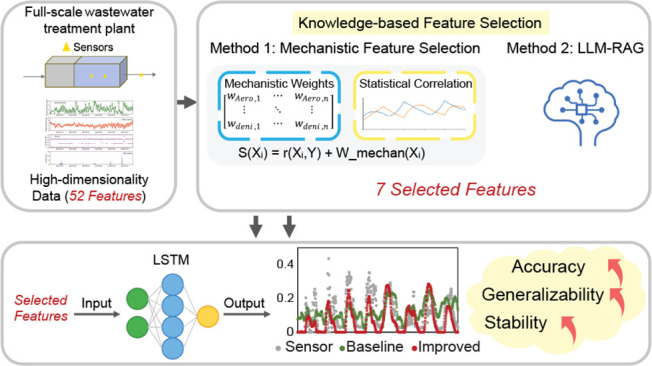

## 1 Introduction

Wastewater treatment modeling has become increasingly crucial for operational optimization, regulatory compliance, and addressing environmental concerns. Traditional mechanistic models, however, face significant limitations due to incomplete understanding of complex reaction mechanisms and microbial interactions, particularly when characterizing emerging issues that attract growing public attention such as emerging contaminants and GHG emissions.^1–4^ In recent years, the role of wastewater treatment systems has expanded beyond pollution removal toward energy-neutral and carbon-negative infrastructure.^5^ Wastewater treatment plants (WWTPs) are increasingly recognized as resource recovery facilities capable of generating energy, recovering nutrients, and reducing net GHG emissions.^6,7^ This transition places higher demands on process monitoring and optimization, which in turn requires reliable predictive models capable of accurately capturing system dynamics under diverse operational conditions.^8^

While machine learning models demonstrate promise for modeling complex systems, they encounter the ″curse of dimensionality″ challenge, where an abundance of features leads to overfitting and reduced generalizability, ultimately compromising real-world performance.^9,10^ In wastewater treatment systems, this challenge is exacerbated by small-sized data sets which are generated from extensive monitoring and analytical techniques performed on a limited number of samples. Paradoxically, this wealth of data often obscures rather than illuminates the fundamental mechanistic relationships governing system performance.^11–13^

Recent research has attempted to address high-dimensionality challenges through sophisticated model architectures, including deep learning approaches with convolutional neural networks, graph-based representations, and attention mechanisms designed to capture complex spatiotemporal dependencies in wastewater treatment data.^14,15^ While these innovations theoretically enable more effective navigation of high-dimensionality spaces by automatically identifying relevant features without explicit selection, practical limitations remain. Most wastewater facilities typically generate temporally sparse data sets due to monitoring constraints. Consequently, despite their theoretical advantages, increased architectural complexity and additional input features often lead to deteriorating rather than improved predictive accuracy.^16^ Some investigators have attempted to overcome these limitations by utilizing models that learn from mathematical equations and mechanistic descriptions of known principles to approach supervised learning tasks, offering potential advantages in data-sparse conditions.^11,17^

It is recognized that feature selection can provide a more effective pathway to enhanced model performance than architectural sophistication alone.^18,19^ Through use of rigorous methodologies for identification of priority input parameters, models can achieve superior predictive performance while maintaining reasonable complexity and computational efficiency. However, traditional statistical feature selection methods, such as principal component analysis, lack domain context in wastewater modeling, potentially leading to the selection of features with high statistical significance but limited practical relevance.^20^ Therefore, in this study, we propose that knowledge-guided feature selection based on wastewater domain knowledge offers an effective approach for constraining the model hypothesis space, potentially improving both predictive accuracy and generalizability.

Furthermore, large language models (LLMs) combined with retrieval-augmented generation (RAG) present new opportunities for knowledge-based feature selection by enabling systematic synthesis of domain knowledge from scientific literature.^21–23^ These systems can identify feature relevance across a broad body of evidence, potentially capturing relationships that individual experts might overlook due to limited literature exposure.^24^ Integrating LLM-RAG-based knowledge extraction with traditional expert knowledge allows feature selection to benefit from both human intuition and machine-enabled literature synthesis.

To address emerging wastewater treatment modeling challenges, we present in this study a novel knowledge-based feature selection framework. In this study, feature selection is treated as a key intermediate step in the modeling workflow, aimed at optimizing the input space and improving predictive performance and generalizability. The framework is demonstrated through a case study of nitrous oxide (N_2_O) emissions prediction at two full-scale wastewater treatment plants. N_2_O, a potent GHG, is generated in wastewater treatment simultaneously from multiple highly dynamic biological processes governed, in different ways, by numerous environmental factors.^25^ It is precisely the high-dimensional challenge that confounds purely data-driven approaches and provides an ideal test case for this new methodology. We systematically compare architecture-based strategies, including attention-enhanced deep learning models, with two knowledge-based approaches: (1) knowledge-based feature selection integrating mechanistic understanding with statistical correlation analysis and (2) LLM-RAG-augmented feature selection leveraging scientific literature synthesis. Model performance is evaluated not only in terms of predictive accuracy but also with respect to overfitting risk and robustness under distributional shift, including temporal transfer across seasonal conditions and external generalizability across data sets from different treatment plants. Through this design, we assess whether improving feature quality, rather than increasing model complexity alone, provides a more effective pathway toward robust wastewater treatment modeling.

## 2 Materials and Methods

### 2.1 Data Acquisition and Preprocessing

#### 2.1.1 Study Site Characteristics

Comprehensive monitoring was conducted at two full-scale wastewater treatment plants (WWTPs) located in Queensland, Australia, termed WWTP-A and WWTP-B.

WWTP-A has a design treatment capacity of 20 ML day^–1^ and employs a biological nutrient removal configuration consisting of sequential anoxic and aerobic zones followed by secondary clarification. The plant operates with mechanical surface aerators in the aeration basins. The facility includes six parallel biological treatment basins, each consisting of an anoxic zone (tank 1) followed by an aerobic zone divided into two compartments (tanks 2 and 3) equipped with surface aerators. Monitoring campaigns primarily focus on one representative basin.

WWTP-B has a design treatment capacity of 30 ML·d^–1^ and employs a five-stage Bardenpho process. It utilizes a multicompartment biological reactor consisting of 12 sequential tanks, including anaerobic, aerobic, postanoxic, and reaeration zones, arranged in parallel treatment trains. The reactor configuration allows staged biological nutrient removal through multiple redox environments. Monitoring campaigns focused on one representative treatment train.

#### 2.1.2 Data Sets and Pretreatments

Different data pretreatment methods were implemented for the data sets used in this study. Three data sets were employed to evaluate the proposed modeling framework, including two data sets from WWTP-A and one data set from WWTP-B (Table 1).

**Table 1:** Summary of Data Sets and Monitored Raw Readings

**Data set**	**WWTP**	**Monitoring period**	**Raw readings (SCADA variables)**
A1	WWTP-A	May 2024 – July 2024	N_2_O concentration, RAS_CDE, RAS_F, Aeration Power2, WAS, InflowRate, DO, Aeration On/Off status, effluent ammonia, effluent nitrate
		9–16 August 2024	
A2	WWTP-A	5–15 December 2024	
B1	WWTP-B	October 2025 – November 2025	Influent flow, WAS flow, RAS pump flows, internal recycle pump speeds, aeration airflow (cells 1–3 and reaeration cell), DO (cells 1–3), reaeration DO, temperature, ammonia, nitrate, N_2_O concentration

Data set A1 was collected from WWTP-A during a monitoring campaign from 25 May 2024 to 16 August 2024. Data set A2 was obtained from the same treatment plant during a second monitoring campaign from 5 to 15 December 2024, representing a different seasonal period. Both data sets were acquired from the SCADA (supervisory control and data acquisition) system and included the same set of monitored variables (Table 1). The recorded parameters included dissolved N_2_O concentrations, return activated sludge (RAS) parameters (RAS_CDE and RAS_F), nitrogen indicators (ammonium and nitrate concentrations), operational metrics (aeration power2, WAS, inflow rate, DO, and aeration on/off status), and effluent quality indicators (effluent ammonia and effluent nitrate concentrations).

Data set B1 was obtained from WWTP-B during a monitoring campaign from October to November 2025. The SCADA system recorded a range of operational and process variables, including influent and sludge flow rates, RAS pump flows, internal recycle pump speeds (A-cycle flow rates), aeration airflow rates in different aeration cells 1–3, dissolved oxygen concentrations in aeration tanks 1–3, pH in the reactor effluent, temperature in aeration tank 1, nitrogen indicators (ammonia and nitrate), and N_2_O concentrations measured by in situ probes.

The original SCADA data were recorded at varying time intervals. To reduce computational complexity and better capture relevant process dynamics, the SCADA data from all data sets were resampled to 15 min intervals by calculating the mean value of each parameter within each 15 min time window.

In addition, hourly precipitation data corresponding to the catchment areas of both wastewater treatment plants were obtained from the CHRS Data Portal (PERSIANN-CCS). Linear interpolation was applied to align the rainfall data with the 15 min timestamps of the aggregated SCADA data sets, enabling integrated analysis of precipitation effects on treatment performance and N_2_O emissions.

#### 2.1.3 Feature Engineering

To further investigate the intermediate reaction processes contributing to N_2_O emissions, we implemented an Activated Sludge Model - N_2_O (ASM-N_2_O) that was calibrated specifically for our system using the first 2 weeks of data.^26^ To prevent data leakage in the downstream deep learning models (section 2.2), the calibration period was confined to data that falls within the training set, ensuring that no information from the validation or test sets was used during model calibration. This model incorporates the bioconversion pathways and kinetics described by Pocquet et al. for AOB-mediated N_2_O production via nitrifier nitrification (NN) and nitrifier denitrification (ND), integrated with the four-step heterotrophic denitrification model from Hiatt and Grady.^27,28^ The model was separately calibrated using operational data from WWTP-A and WWTP-B, with particular attention to dissolved oxygen concentration profiles and nitrogen species transformations. The calibrated model generated a comprehensive set of state variables describing the biological nitrogen transformation processes, including dissolved gas concentrations (N_2_ and N_2_O), dissolved nitrogen species (NH_4_^+^, NH_2_OH, NO, NO_2_^–^, and NO_3_^–^), dissolved oxygen levels, readily biodegradable substrate concentrations, and biomass concentrations (X_AOB, X_H, X_I, and X_NOB). The simulated temporal dynamics of these variables are presented in SI section 2.

### 2.2 Deep Learning Model Development and Implementation

For baseline performance assessment, we initially implemented a standard long short-term memory (LSTM) neural network architecture, which has demonstrated effectiveness in capturing temporal dependencies in environmental time series data.^29^ For baseline performance assessment, we implemented a two-layer LSTM architecture. The baseline model comprised two stacked LSTM layers (32 units each), with each layer followed by batch normalization and dropout regularization (rate = 0.2) and a final linear dense output layer.

Building upon this foundation, we progressively increased the model architecture complexity through two primary mechanisms:

1. **Architectural Enhancement:**
  Building upon the two-layer baseline, we conducted systematic hyperparameter optimization to search across architectures of varying depth and configuration. The number of LSTM layers, unit counts per layer, and regularization parameters was jointly optimized using the Optuna framework,^30^ allowing the search to identify the best-performing architecture without manual exploration.
2. **Attention–LSTM Model:**
  To enhance model interpretability and performance, we evaluated an Attention–LSTM architecture designed to capture complex dependencies in the data. This model applies multihead self-attention across the feature dimension prior to temporal modeling via LSTM layers. By prioritizing informative features early in the processing pipeline, this architecture enhances model flexibility and robustness, particularly when dealing with high-dimensionality data sets.
  The attention mechanisms were implemented as multihead self-attention layers following the formulation
$${\mathrm{head}}_{i}=\mathrm{Attention}\left(QW_{i}^{Q},KW_{i}^{K},VW_{i}^{V}\right)$$
$$\mathrm{MultiHead}\left(Q,K,V\right)=\mathrm{Concat}\left({\mathrm{head}}_{1},...,{\mathrm{head}}_{h}\right)W^{O}$$
  where *Q*, *K*, and *V* represent the query, key, and value matrices derived from the input representations. The matrices *W*_*i*_^*Q*^, *W*_*i*_^*K*^, *W*_*i*_^*V*^, and *W* ^*O*^ are learnable projection matrices that transform the input representations into query, key, value, and output spaces, respectively.^31^
3. **CNN–LSTM Model:**
  In addition, a one-dimensional Convolutional Neural Network–LSTM (CNN-LSTM) hybrid architecture was evaluated. The CNN component applies temporal convolutional filters to extract local short-range patterns from the input sequence, after which the LSTM layers capture long-range dependencies from these condensed representations. This hierarchical feature extraction strategy has been shown to improve predictive performance in multivariate time series tasks.^32^

To ensure rigorous and reproducible model development, all architectures underwent systematic hyperparameter optimization using the Optuna framework with Tree-structured Parzen Estimator (TPE) sampling and Hyperband pruning.^30^ For each architecture, 100 optimization trials were conducted, with an initial random sampling phase of 15 trials to establish a diverse starting population before TPE-guided search commenced. Hyperparameter optimization was performed for each model architecture, including both architectural and training-related parameters. The complete hyperparameter search space and the optimal configurations identified for each model are summarized in Tables S4 and S5 in the Supporting Information. Each trial was evaluated using the validation mean absolute error (MAE) computed on inverse-transformed predictions to reflect true physical units. The optimal hyperparameter configuration identified for each architecture was subsequently used for the final model training.

All models were implemented in TensorFlow using the Keras API and trained with the Huber loss and Adam optimization, together with adaptive learning rate reduction and early stopping to prevent overfitting. For Data Set A1, the 51-day in-distribution data set was split chronologically into 30 days for training, 10 days for validation, and 11 days for testing. For data set B1, the 39-day data set was split into 25 days for training, 5 days for validation, and 9 days for testing. In both cases, the chronological order was preserved to maintain temporal integrity. For robust comparison, each model configuration was trained 20 times with different random initializations, and performance metrics (MAE and R^2^) were averaged to account for the training stochasticity. The computational requirements of each component in the proposed framework are summarized in Table S6.

### 2.3 Knowledge-Based Feature Selection Framework

A knowledge-based feature selection framework was applied independently to optimize the input variable space before downstream model development and hyperparameter optimization. This procedure followed a structured workflow to identify the most relevant variables for predicting N_2_O emissions while balancing mechanistic understanding with statistical correlation:

1. Mechanistic Importance-Weighting
  We first categorized features based on their functional roles within the biokinetic equations. Subsequently, Mechanistic Importance scores (MI-scores) were assigned to each feature according to its theoretical relevance to established mechanistic pathways, including kinetic equations and mass balance considerations (detailed in SI Section 3). These scores range from near-zero (indicating minimal relevance) to 1.0 (indicating maximum relevance) and reflect the theoretical contribution of each variable to N_2_O production as informed by well-established biochemical reaction mechanisms.
2. Process Group Categorization
  Features were grouped into functional categories corresponding to their biological roles in the nitrogen removal processes. The AOB pathway includes ammonia, hydroxylamine, nitrite, and relevant autotrophic biomass concentrations; the NOB pathway includes nitrite, nitrate, and NOB biomass concentrations; heterotrophic denitrification includes nitrogen oxides and heterotrophic biomass concentrations; and other parameters include operational controls, real-time sensors, and weather-related variables.
3. Temporal Correlation Analysis
  A time-matching correlation analysis was conducted using the ccf (cross-correlation function) from the statsmodels.tsa.stattools python package, which computes Pearson correlation coefficients between two time series at multiple time lags. For each process variable (e.g., dissolved oxygen, influent ammonia concentrations), we calculated cross-correlations with N_2_O emissions over a ±4-h window using 15 min resolution data. This enabled identification of leading or lagging relationships, indicating whether changes in a feature tend to precede or follow N_2_O fluctuations. The maximum absolute correlation within this window was recorded as the Statistical Correlation score (SC-score), representing the temporal predictive strength of each variable.
4. Redundancy Elimination
  A feature similarity matrix was constructed by calculating pairwise time-matching Pearson correlation coefficients between normalized feature time series. Features were also grouped by shared process roles (e.g., nitrogen transformation or aeration control), and intragroup pairs were assigned elevated similarity scores to account for functional redundancy.
5. Final Feature Selection
  Features were ranked based on a weighted combination of their SC and MI scores. Prior to combination, both scores were linearly normalized to a common scale to ensure a comparability.
CombinedScore=SC‐score+MI‐score
  Redundant features (similarity > 0.85) were removed iteratively, retaining the feature with the higher combined score. The threshold of 0.85 was selected as a conservative cutoff to capture strong interfeature redundancy and may be adjusted depending on data characteristics.

For comparison, LASSO regularization and SHAP-based feature importance analyses were independently applied as benchmark feature selection methods. Detailed procedures and results are provided in section 7 of the Supporting Information.

### 2.4 LLM-RAG Feature Selection Framework

To support feature evaluation and selection for the data-driven modeling, a RAG framework was implemented to integrate domain knowledge from scientific literature with the operational data set. The framework was built using the PaperQA library,^33–35^ which enables large language models to retrieve and reason over indexed research documents. Three LLMs were used as the question-answering models in the RAG framework, representing different architectures, training methodologies, and knowledge cutoff dates, as summarized in Table 2.

**Table 2:** Summary of LLMs with Corresponding Developers and Knowledge Cutoffs

**Model**	**Developer**	**Knowledge Cutoff**
GPT-5.2	OpenAI	December 2025
Gemini 2.5 Flash	Google	June 2025
Claude 4.6 Sonnet	Anthropic	February 2026

A local corpus of wastewater treatment and N_2_O emission studies, including 520 N_2_O-related articles from leading journals, was compiled and indexed using vector embeddings generated with the OpenAI text-embedding-3-small model, enabling a semantic similarity search across the literature database. For reasoning and response generation, a multi-LLM framework incorporating GPT-5.2, Claude Sonnet 4.6, and Gemini 2.5 Flash models was employed to leverage complementary reasoning capabilities across different architectures.

When the feature selection query (provided in the Supporting Information) together with the anonymized data set was submitted to the framework, the system first interpreted the data set through structured summaries, including feature descriptions, statistical characteristics, missing-value analysis, and correlations between process variables and the target variable (N_2_O concentration). Prior to submission, all plant-identifying information, site names, and operationally sensitive metadata were removed from the data set to ensure data confidentiality. Relevant passages were then retrieved from the indexed literature corpus to provide an additional domain knowledge.

On the basis of the combined data set context and retrieved literature information, the framework generated the final feature selection results, which were subsequently used as inputs to the trained predictive model.

## 3 Results and Discussion

### 3.1 Deeper and Attention-Based Models Improve Accuracy but Show Limited Generalizability

To systematically evaluate modeling options under the “small-sample, high-dimensional” setting (sample-to-feature ratio ≈ 76:1), two widely adopted strategies were examined: (1) searching for deeper architectures capable of capturing temporal dynamics and (2) incorporating attention mechanisms.

In the initial experiments, a two-layer LSTM was employed for prediction, but its performance was limited, achieving a mean test MAE of 0.0665 (σ = 0.0110) and a mean R^2^ of 0.119 across 20 runs. A CNN–LSTM hybrid was also evaluated but yielded only marginal improvement (MAE = 0.0599, R^2^ = 0.206) and was not pursued further. To assess whether deeper architecture could better capture temporal patterns, we conducted a systematic hyperparameter search over key architectural and training parameters, with regularization applied to mitigate overfitting under limited samples. The resulting five-layer LSTM achieved a mean test MAE of 0.0567 (σ = 0.0065) and a mean R^2^ of 0.2263 (σ = 0.1843), a modest improvement over the two-layer baseline (0.0665). Incorporating attention mechanisms led to further improvements. Two variants were examined: Attention-LSTM, which employs feature-wise attention to reweight input features before sequential processing, and LSTM-Attention, which applies temporal attention after LSTM-based temporal encoding. Attention-LSTM achieves a mean test MAE of 0.0326 (R^2^ = 0.712), outperforming LSTM-Attention (MAE = 0.0478, R^2^ = 0.465) by 31.8%.

These results show that all evaluated model variants, including deeper LSTM, LSTM-attention, and attention-LSTM, improve predictive performance relative to the baseline. The performance gain observed in the five-layer LSTM may be attributed to its enhanced capacity to capture longer-range temporal dependencies, which has been reported in prior studies on recurrent architectures.^36^ In contrast, the two attention variants provide a controlled comparison that isolates the effect of the attention placement. The observed performance difference between Attention-LSTM and LSTM-Attention suggests that the stage at which attention is applied influences how feature information is extracted and used. Reweighting inputs prior to sequential processing appears more effective than applying attention to already encoded representations, indicating that the quality and relevance of input features may play a particularly important role in this setting. This is consistent with findings in high-dimensional time series modeling, where input feature quality has been shown to substantially affect model performance, and where early stage feature weighting has been found to improve information utilization under limited sample conditions.^31,37^

Despite these improvements in test performance, their robustness remains uncertain. As shown in Figure 1b, all architectures exhibit overfitting to varying degrees, with validation-to-training MAE ratios ranging from 1.26x (LSTM-5-Layers) to 1.66x (Attention-LSTM), indicating that the gap between training and validation performance persists across all evaluated configurations. To further assess generalization under distributional shift, models were evaluated on an out-of-distribution data set collected during a high-flow period in August, one month after the training and validation period, when the influent flow rate (64.04 L/s) substantially exceeds the training mean (52.71 L/s). As shown in Figure 1d, all architectures fail markedly under these conditions, with R^2^ values turning negative across all models (Baseline: −0.017, LSTM-5-Layers: −0.084, LSTM-Attention: −0.199, Attention-LSTM: −0.087) and MAE values substantially exceeding those observed in July. This collapse in performance indicates that the high-flow event introduces feature combinations that are underrepresented in the training data, forcing the model to extrapolate beyond the learned feature distribution and disrupting the learned input–output relationships. Similar behavior has been reported in data-driven hydrological modeling and sequence learning tasks under distributional shift.^38,39^ The strong in-distribution performance of Attention-LSTM may therefore partly reflect overfitting to the statistical characteristics of the July period rather than robust generalization.^40^

**Figure 1:**
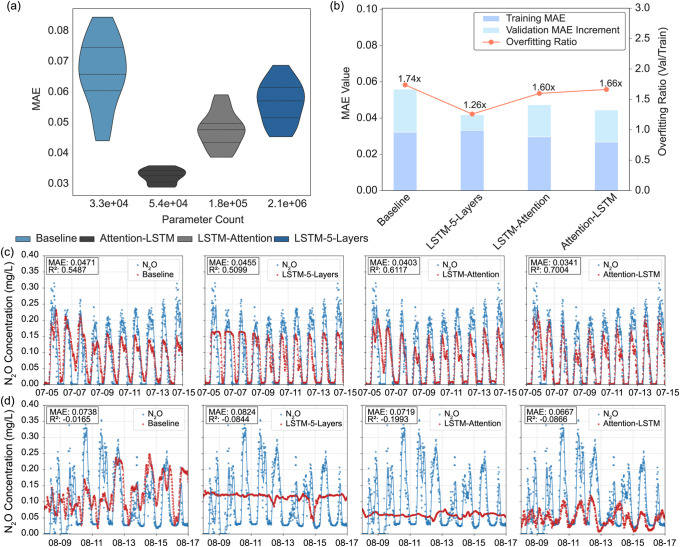
Model performance comparison under small-sample, high-dimensional conditions. (a) Test MAE distributions show progressive improvement from the baseline LSTM to deeper and attention-based models, with Attention-LSTM achieving the lowest error and variance. (b) Training–validation comparison indicates reduced error but increased overfitting in higher-capacity models. (c) Under in-distribution conditions (July), Attention-LSTM best captures N_2_O dynamics. (d) Under distributional shift (August high-flow), all models degrade markedly, failing to reproduce observed variability.

Taken together, these results indicate that while increasing model depth and incorporating attention mechanisms can improve predictive accuracy, they also introduce challenges such as overfitting under limited data. A likely contributing factor is the high sample-to-feature ratio (≈76:1), under which it becomes difficult to reliably identify and prioritize informative features. As a result, the model may learn spurious patterns rather than stable relationships, leading to reduced robustness, particularly under distributional shift. These findings suggest that further gains are unlikely to be achieved through architectural modifications alone. Instead, improving the quality of the input space, by reducing feature redundancy and enhancing the signal-to-noise ratio, may be essential for achieving stable and generalizable performance across varying operating conditions.

### 3.2 Expert-Guided Feature Selection Improves Accuracy and Generalizability

To address the generalization limitations identified in section 3.1, we developed a knowledge-based feature selection approach that simultaneously incorporates statistical correlation and mechanistic importance to reduce the input feature space (Figure 2a). As shown in Figure 2c, our analysis revealed several features with a high statistical correlation to Tank 3 N_2_O emissions. The top statistically correlated features included NO-tank3 (1.0), N_2_-tank1 (0.983), and NO-tank2 (0.982). These strong correlations can be partially explained by the fact that nitric oxide is a direct biochemical precursor of N_2_O in both nitrifier and heterotrophic denitrification pathways, while N_2_ levels often reflect the extent of denitrification and may covary with intermediate gases under transient conditions.

**Figure 2:**
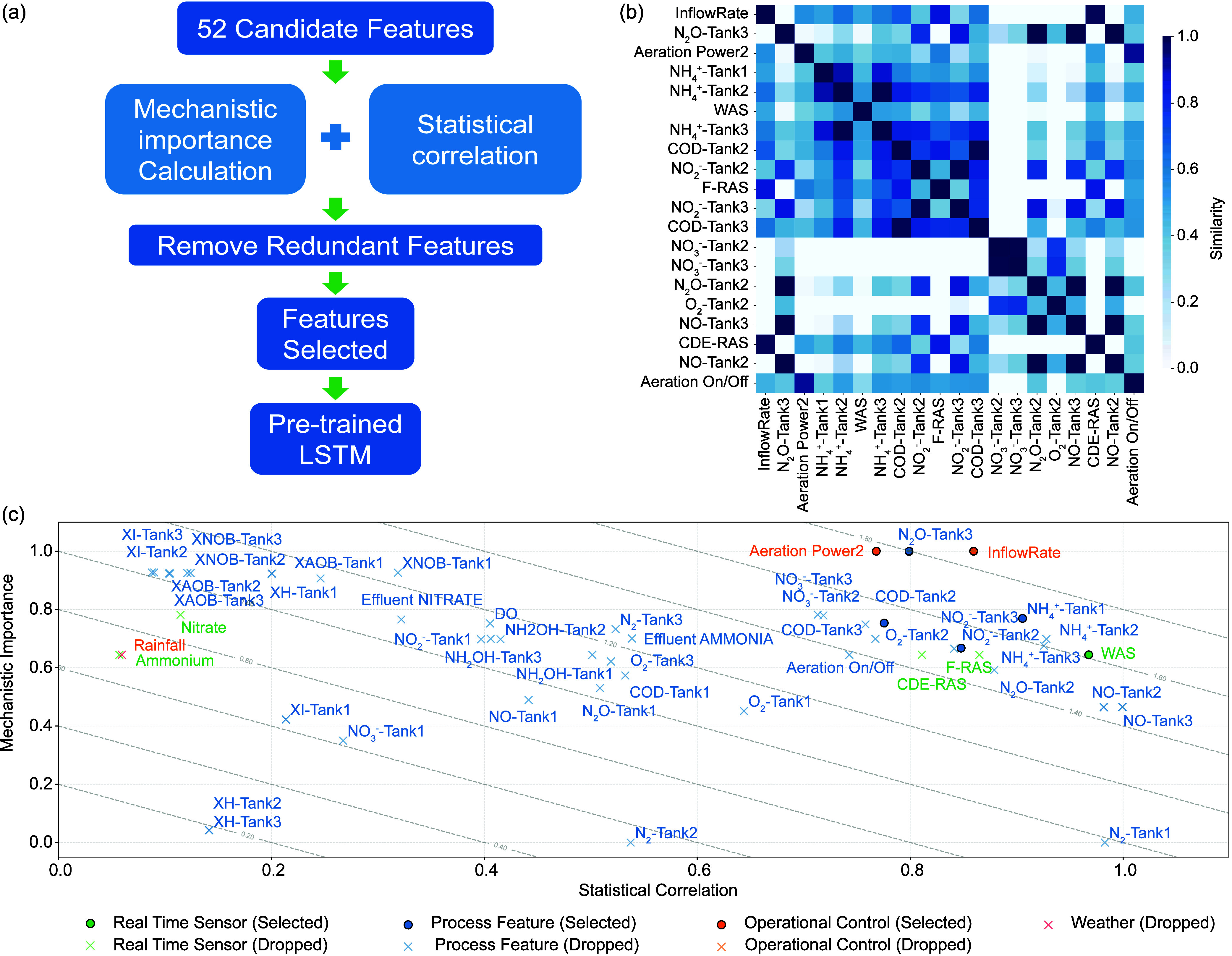
Knowledge-based feature selection framework for N_2_O emissions prediction: (a) Schematic overview of the proposed framework, integrating mechanistic importance and statistical correlation to reduce 52 candidate features to a final selected subset for input to the pretrained LSTM model; (b) Similarity matrix of monitored parameters showing correlation patterns. (c) Statistical correlation vs mechanistic importance scores for different parameter categories; dashed line indicates equal contribution from both.

However, statistical correlation primarily reflects direct linear relationships among observable variables and is often susceptible to data-collection noise and sample-distribution biases. Therefore, we incorporated the mechanistic importance as a complementary selection criterion. Mechanistic importance, grounded in process knowledge, captures both causal and indirect relationships within the system. Interestingly, some features demonstrated high mechanistic importance, despite moderate statistical correlations. For instance, InflowRate, N_2_O-Tank3 (simulated N_2_O concentration), and Aeration Power2 all received maximum mechanistic importance scores (1.0), suggesting their significance in N_2_O production pathways according to the process understanding. The high mechanistic importance of InflowRate can be attributed to its direct influence on the hydraulic retention time and substrate availability, while Aeration Power2 controls dissolved oxygen levels that significantly impact nitrification and denitrification rates.

To mitigate feature redundancy and avoid multicollinearity, parameter instability, and reduced generalizability, we performed a similarity analysis of the normalized feature time series (Figure 2b). Specifically, we observed that several features, such as NH_4_^+^-Tank1 and NH_4_^+^-Tank2, exhibited near-identical temporal patterns after normalization. This pattern similarity likely arises from their shared mechanistic roles in the nitrification process, where NH_4_^+^ concentrations are governed by similar operational conditions (e.g., inflow ammonia concentration, aeration intensity). This type of redundancy, if left unaddressed, can introduce instability in model training and reduce generalization performance.^41^ We defined a similarity threshold of 0.85 using Pearson’s correlation. Features exceeding this threshold were considered redundant, and we retained the one with the higher combined statistical and mechanistic score. For example, NH_4_^+^-Tank2 was removed due to high similarity (0.906) with NH_4_^+^-Tank1, and NO_2_^–^-Tank3 was excluded due to overlap with N_2_O-Tank3 (similarity score: 0.865).

After evaluating multiple sample-to-feature ratios and testing feature subsets of varying sizes, we ultimately selected seven features: inflow rate, N_2_O-tank3, aeration power2, NH_4_^+^-tank1, WAS flow, COD-tank2, and NO_2_-tank2. These features collectively represent the core operational controls, nitrogen transformation indicators, and organic loading conditions relevant to N_2_O dynamics. InflowRate, Aeration Power2, and WAS flow reflect key process operations; NH_4_^+^-Tank1 and NO_2_^–^-Tank2 capture critical steps in nitrification and denitrification; COD-Tank2 serves as a proxy for organic carbon availability governing denitrification; and N_2_O-Tank3 acts as a direct proxy for the target variable in prediction. This selection balances mechanistic relevance and statistical efficiency while reducing redundancy.

To further evaluate the effectiveness of the proposed feature selection strategy, the reduced feature subset was first applied with the basic 2-layer LSTM model structure (FS-basic-LSTM) introduced in Section 3.1. The FS-basic-LSTM trained on the selected features achieves strong predictive performance under in-distribution conditions, with a mean test MAE of 0.0367 (σ = 0.0032) and a mean R^2^ of 0.6919. This represents a significant improvement over the baseline model (*p* < 0.001), the five-layer LSTM model (*p* < 0.001), and the LSTM-Attention model (*p* < 0.001) (Table S7). The R^2^ is comparable to that of the Attention-LSTM, with no statistic difference. In the best-performing run, the model achieves an R^2^ of 0.77, exceeding the best result obtained by the Attention-LSTM.

Using the same hyperparameter search strategy as in Section 3.1, model architecture optimization was further conducted on the selected feature set. Notably, the optimal configuration corresponds to a relatively straightforward 1-layer architecture (FS-tuned-LSTM). After tuning, the model exhibits improved stability but only marginal gains in predictive accuracy (mean MAE = 0.0330, σ = 0.0015; mean R^2^ = 0.7225). Both feature-selected models, as shown in Figure 3a, more effectively capture the diurnal patterns of N_2_O emissions compared to their full-feature counterparts. This improvement can be attributed to the reduction of multicollinearity and noise in the input space.^42^ Besides, the selected feature subset retained stable predictive performance across LSTM architectures with varying depths (Tables S8 and S9), indicating that the selected feature subset remained robust under different model configurations.

**Figure 3:**
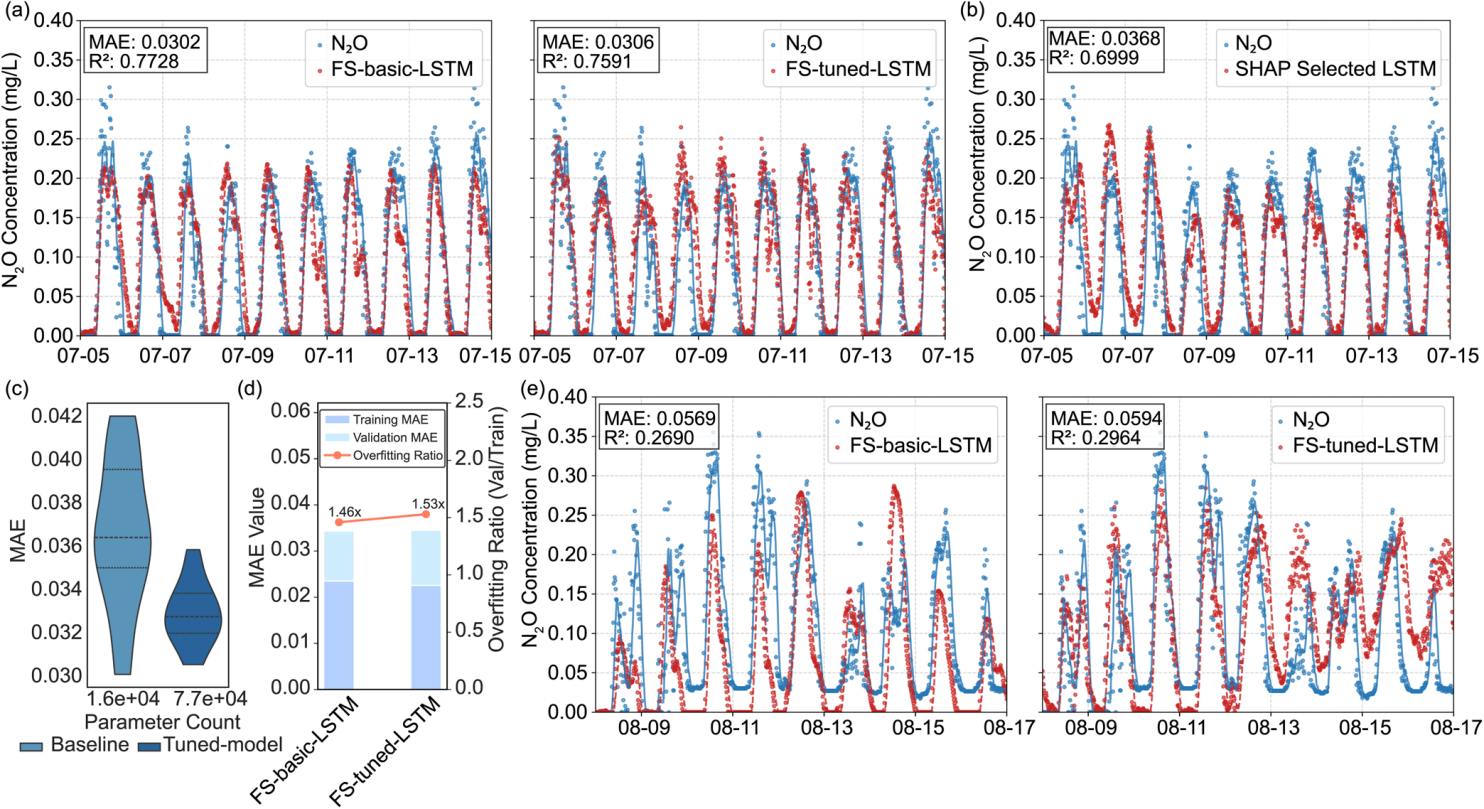
Performance of feature-selected LSTM models for N_2_O prediction: (a) measured vs predicted time series under in-distribution conditions (July) for baseline and tuned models; (b) same comparison for the SHAP-selected tuned model; (c) MAE distribution across 20 runs as a function of model size; (d) overfitting analysis based on training and validation MAE and their ratio; (e) performance under out-of-distribution conditions (August high-flow). FS-basic-LSTM: 2-layer basic LSTM model with selected features as input; FS-tuned-LSTM: hyperparameter-tuned 1-layer LSTM model with selected features as input.

To assess whether the performance gains arise from feature reduction alone or from the proposed framework, we first applied LASSO regularization to select features based on coefficient shrinkage and subsequently applied SHAP to select seven features from the tuned 5-layer LSTM for a controlled comparison. However, the resulting model still underperformed compared to the FS models (Figure 3b; Tables S11 and S13). In contrast, mechanistic feature selection retains the most informative predictors, enabling a simpler yet more informative feature space for stable and efficient learning of system dynamics.^43^ These results indicate that model performance is primarily determined by feature quality rather than model architecture, leading to reduced sensitivity to hyperparameter settings and improved stability in practice.^44^

In addition to predictive accuracy, model overfitting was evaluated using the validation-to-training MAE ratio. As shown in Figure 3d, the FS-basic-LSTM model exhibits an overfitting ratio of approximately 1.46×, while FS-tuned-LSTM shows a slightly higher ratio of 1.53× (Figure 3d). Nevertheless, the overall magnitude of overfitting is reduced compared to the results obtained using the full feature set in Section 3.1.

Robustness under distributional shift was further evaluated using the out-of-distribution August data set. After feature selection, both the FS-basic-LSTM and FS-tuned-LSTM achieve positive R^2^ values (0.2690 and 0.2964, respectively). Although this data set lies outside the range of the training data, both models are able to capture the majority of temporal fluctuations in N_2_O emissions, including the timing and general shape of peak events, suggesting that the selected features effectively preserve the dominant system dynamics (Figure 3e). Importantly, prediction accuracy remains high across most of the August period and only begins to deteriorate when inflow conditions increase far beyond the training range. Specifically, when the inflow rate reaches 82.62 L/s (August 14–16), representing a 56.74% increase relative to the mean influent flow in the training data set, model performance shows a noticeable decline. This indicates that predictive performance may be limited when extrapolating beyond the training domain and suggests that future applications could benefit from incorporating additional data or adaptive retraining strategies.^45^

Overall, these results indicate that the main improvement in model performance arises from the knowledge-guided reduction of the input space. By removing redundant and less informative variables, the selected feature set enables the model to learn more stable relationships from limited data, resulting in improved predictive accuracy and better retention of predictive skill under a distributional shift. Compared with the deeper and attention-based full-feature models, the feature-selected models more effectively preserve the dominant temporal dynamics of N_2_O emissions under the shifted conditions.This suggests that, in small-sample, high-dimensional wastewater treatment modeling, mechanistically informed feature selection may be more important than architectural complexity for achieving robust generalization.^46^

### 3.3 LLM-Assisted Feature Selection Provides a Viable Alternative

To evaluate whether LLM-RAG-based feature selection can serve as a viable alternative to expert knowledge, we constructed a RAG framework that synthesizes domain knowledge from scientific literature to guide feature selection without direct expert involvement. A total of 15 experimental runs were conducted, with each of the three LLMs evaluated five times, and seven features were selected in each run. Across all runs, 14 unique features were selected in total (Figure 4a). DO and InflowRate were consistently chosen (100%), followed by N_2_O-Tank3 (80%) and Aeration Power2 (67%), together accounting for 46% of all selections (Figure 4a). The repeated selection of these variables indicates that the framework converges on a stable set of mechanistically relevant predictors rather than selecting features arbitrarily. In addition, a comparison across three LLMs revealed only minor differences in feature frequencies (Figure 4a). All models consistently selected DO and InflowRate in all rounds, with closely aligned frequencies for other core variables such as N_2_O-tank3. Minor variations in less frequently selected features did not affect the overall composition or the interpretability of the feature set. This suggests that output stability is driven by the RAG retrieval mechanism rather than model-specific preferences. The complete feature selection reports generated by each LLM across all five runs are provided in section 9 of the Supporting Information.

**Figure 4:**
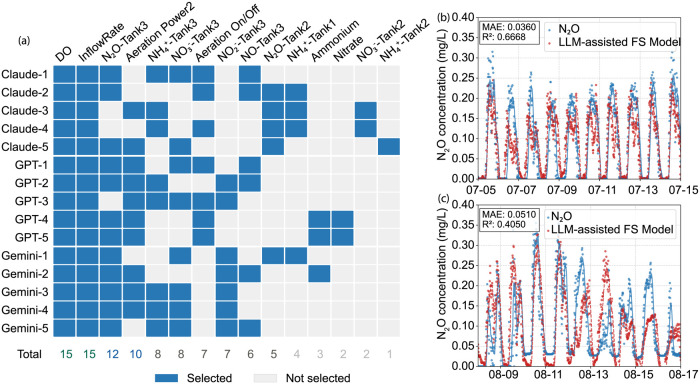
LLM-assisted feature selection results and model performance: (a) feature selection matrix across 15 experimental rounds for three LLMs (Claude, GPT, and Gemini), showing selected and unselected features per round with total selection frequency indicated below; (b) time series comparison of predicted versus measured N_2_O concentrations under in-distribution conditions (July) using the LLM-assisted selected feature set; (c) time series comparison under out-of-distribution conditions (August high-flow period).

Seven features including DO, InflowRate, N_2_O-Tank3, Aeration Power2, NH_4_^+^-Tank1, NO_2_^–^-Tank3, and NO_3_^–^-Tank3 are selected. This exact set was recovered in 2 of 15 rounds, and all seven features ranked among the eight most frequently selected across the 14 candidates. Those core features are directly linked to N_2_O production pathways: DO regulates AOB-mediated nitrification, inflow rate reflects hydraulic and substrate dynamics, N_2_O-Tank3 provides autoregressive temporal information, and aeration power2 captures aeration control effects. Additional features include ammonium, nitrite, and nitrate concentrations, representing nitrogen transformation states that are relevant to N_2_O production.

In comparison with the expert-based feature set, three core features (InflowRate, N_2_O-Tank3, and Aeration Power2) are consistently identified by LLM-RAG, while NH_4_^+^-Tank1 appears to be less frequent. In contrast, NO_2_–Tank2, WAS, and COD-Tank2 are not selected under the RAG approach, marking the main divergence. This may be because WAS and COD-Tank2 are operational variables that are less explicitly discussed in N_2_O modeling literature, limiting their visibility in RAG retrieval. This difference likely reflects a key limitation of the LLM-RAG approach. While it can synthesize general mechanistic knowledge from the literature, it does not fully capture the plant-specific operational context, process interactions, and local conditions that shape the feature relevance in a particular treatment system. As a result, variables that are important under the specific conditions of this plant may be underprioritized, even if they are mechanistically meaningful as reflected in data sets.

The final LLM-selected features were used to train a single-layer LSTM model with optimized hyperparameters. The model achieved a mean test MAE of 0.0412 (σ = 0.0025) and a mean R^2^ of 0.596 across 20 runs, with the best run reaching an MAE of 0.0366 and an R^2^ of 0.666 (Figure 4b). This performance exceeds that of the five-layer LSTM and LSTM-Attention models and approaches that of the Attention-LSTM. Compared with the expert-guided feature selection models, the MAE from the LLM-feature model is higher (0.0302 vs 0.0412, 36% higher) and R^2^ is lower by 0.096. The results indicate that the LLM-RAG framework realized a substantial portion of the performance gains from feature selection without direct expert input.

Under the distributional shift during the August high-flow period, the model retained an R^2^ of 0.405 and a test MAE of 0.051. The predictive accuracy remains reasonable during most of the August evaluation window but deteriorates notably during the extreme inflow event of August 14–16. The performance under out-of-distribution conditions confirms that the LLM-RAG-selected features preserve sufficient mechanistic signal to generalize partially beyond the training domain, a meaningful result, given that the feature set was constructed without explicit operational knowledge.

Taken together, these findings suggest that LLM-RAG-based feature selection can provide a viable alternative to expert-guided approaches, particularly when direct domain expertise is unavailable. Its key limitation, however, is that it draws primarily on general knowledge from the literature and, therefore, cannot fully account for the plant-specific operating context that shapes feature relevance in an individual treatment system. Consequently, variables that are important under the specific conditions of a given plant may be underprioritized even when they are mechanistically or operationally meaningful.

### 3.4 Generalizability across Seasons and Treatment Plants

To evaluate the broader applicability of the proposed knowledge-based feature selection framework, we conducted two complementary validation experiments. First, we assessed temporal generalizability using data set A2, which was collected from WWTP-A during a different operational period representing summer conditions (December 2024). In this test, the best-performing model configurations identified in sections 3.1 and 3.2 were directly applied without further retraining or fine-tuning. Second, we assessed cross-plant transferability of the framework using data set B1 from another full-scale WWTP (plant B) with a distinct process configuration. In this case, the feature selection framework was reapplied to the new plant prior to model development.

As shown in Figure 5a, all three models exhibited some degree of performance degradation when evaluated on data set A2, which was associated with a substantially higher influent flow rate (75 L/s) than the training data set. However, the extent of degradation differed markedly among the models. The FS-LSTM model showed a systematic scaling bias, with predictions consistently higher than the observed values. Nevertheless, after normalization (Figure S6), it remained able to capture the dominant temporal dynamic patterns of the N_2_O emissions. In contrast, both the LSTM-attention and attention-LSTM models failed to accurately reproduce these peak events, showing reduced sensitivity to transient fluctuations and a tendency to smooth extreme values. These results suggest that the expert-selected feature set retains predictive relevance under seasonal and hydraulic shifts, more consistent with the underlying biochemical mechanisms governing N_2_O dynamics rather than being overfitted to operational patterns specific to a single monitoring period.

**Figure 5:**
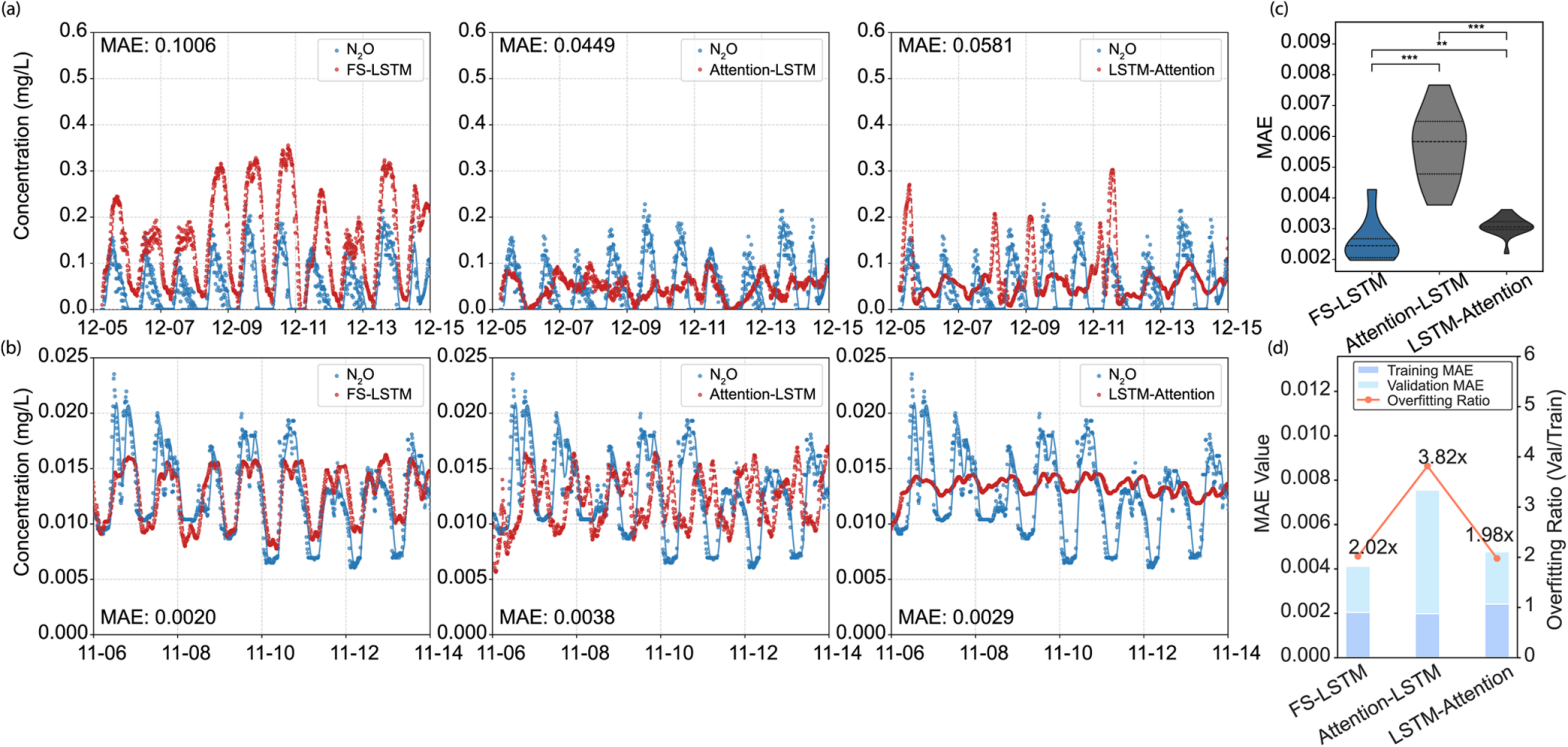
Generalizability assessment of the knowledge-based feature selection framework. (a) Cross-seasonal validation on Data set A2 (WWTP-A, summer period): time series comparison of predicted versus measured N_2_O concentrations using the expert-selected feature set trained on Data set A1. (b) Statistical performance metrics (R^2^, MAE) of LSTM models trained on expert-selected features for WWTP-B (Data set B1) across 20 independent runs. (c) Time series comparison of predicted versus measured N_2_O concentrations for WWTP-B using the expert-selected feature set. (d) Overfitting analysis for WWTP-B showing the validation-to-training MAE ratio for full-feature and feature-selected LSTM models.

The validation using data set B1 was designed to test whether the same feature selection strategy remained effective when transferred to a different treatment system. Plant B consisted of 12 biological tanks and a substantially larger monitoring space, yielding 180 candidate input features when the process variables and sensor measurements were combined. Applying the knowledge-based feature selection framework reduced this set to 7 features including the Airflow Rates Tank 6–8, XH-Tank 5, InflowRate, XH-Tank 6, and COD-Tank2. Compared with the features from plant A, the selected variables in plant B were more strongly associated with denitrification-related processes. This difference highlights the influence of plant-specific configuration and operating conditions on N_2_O dynamics and suggests that feature selection should be re-evaluated when the framework is applied to a new wastewater treatment system.

The plant B feature set was then used as an input to the FS-LSTM, Attention-LSTM, and LSTM-Attention models. Each model was trained 20 times, and the best performing run for each is shown in Figure 5b. Consistent with the results from plant A, the FS-LSTM model more effectively captured the temporal dynamics of N_2_O emissions, whereas both attention-based models showed a reduced ability to reproduce the dynamic fluctuation patterns. The FS-LSTM model also achieved the lowest MAE (0.00260), outperforming the attention-LSTM (0.00568) and LSTM-attention (0.00309) models. Although the overfitting ratios were broadly comparable across models, FS-LSTM exhibited more consistent performance across validation settings (Figure 5d). Such high-dimensional input in Plant B increases the difficulty for the model to learn the true underlying trends, as it may be distracted by noise and spurious correlations, ultimately leading to poorer predictive performance.^38,47,48^ These results collectively demonstrate that the proposed feature selection framework improves practical model generalizability in small-sample, high-dimensional wastewater treatment modeling by reducing irrelevant variation and preserving mechanistically informative inputs.

### 3.5 Implications

This study presents a knowledge-based feature selection framework that integrates mechanistic importance and statistical correlation, augmented by LLM-RAG literature synthesis, for predicting N_2_O emissions in wastewater treatment plants. By reducing 52 candidate variables to seven mechanistically and statistically significant inputs, the expert-guided model achieved a mean R^2^ of 0.723 under small-sample constraints while markedly improving generalizability under out-of-distribution conditions where all full-feature models failed. The results confirm that (i) knowledge-based feature selection addresses the generalization limitations of purely architectural approaches, (ii) feature quality rather than model complexity is the dominant factor controlling performance under small-sample, high-dimensional conditions, and (iii) LLM-RAG-based selection represents a viable alternative to expert-guided approaches, achieving competitive performance without direct expert involvement.

Feature selection is pivotal across fields from genomic risk prediction in bioinformatics^49,50^ and sentiment/text classification in NLP^51,52^ to diagnostic imaging^53^ and credit-risk modeling in finance.^54^ This approach started with simple one-variable filters that tamed the “curse of dimensionality” in high-sensor data sets^55,56^ and evolved into wrapper-style searches that test many candidate subsets in the model loop.^57,58^ With deep learning, construction and selection blurred together: autoencoders, attention maps, and multimodal schemes now sift through images, time-series, and lab results in real time.^59,60^ Despite these proven benefits, most machine learning studies in environmental engineering implement feature selection unsystematically and are not fully aware of its critical role in achieving optimal model performance.^61–64^ Across recent WWTP influent-forecasting and potable-water studies, targeted feature pruning cut input variables by two-thirds, lifted test-set R^2^ by roughly 10–20%, and slashed training time by half, underscoring feature selection’s decisive value in operational water-quality models for researchers and operators.^65–67^

Our approach fuses mechanistic process insight with empirical correlation analysis to construct a feature selection framework that preserves statistical validity and enhances process-aware robustness. The methodology addresses the fundamental challenge in wastewater treatment modeling of how to extract meaningful predictive signals from high-dimensional, sparse data sets where traditional machine learning approaches systematically fail. Incorporating LLM-assisted knowledge extraction into our pipeline represents one of the first applications of LLM-RAG-based feature selection in wastewater engineering. This breakthrough both expands the methodological toolkit and empirically validates our central claim that knowledge-guided selection consistently outperforms purely statistical heuristics. Variables suggested by prompting the LLM with domain knowledge from environmental science and engineering literature delivered competitive predictive accuracy and demonstrated partial generalizability under distributional shift. Beyond N_2_O prediction, the same framework could be extended to other environmental modeling tasks characterized by sparse, high-dimensional, and mechanistically structured data.

Several limitations also warrant future studies. First, the current feature selection process primarily considers the individual contribution of each feature to the target variable. However, in N_2_O production pathways, interactions among features and between feature relevance and model configurations may be significant. Future work should therefore explore interaction-aware feature selection strategies that explicitly account for both feature–feature dependencies and feature–model configuration interactions. Second, the combined feature importance score was based on equal weighting of statistical correlation and mechanistic importance. This 1:1 weighting provides a pragmatic balance for the present system as a proof of concept, but the optimal weighting may vary with data quality, system complexity, and confidence in the underlying mechanistic understanding. For example, in data-scarce scenarios, placing greater emphasis on mechanistic importance may be more appropriate, whereas statistical correlation may play a more dominant role when large, high-quality data sets are available. Therefore, the proposed framework is inherently flexible, and the weighting scheme can be adjusted in future applications to better reflect context-specific conditions. Lastly, due to the discrete nature of the available real-world data, the safe extrapolation bounds of the proposed method could not be systematically defined. Further validation under broader and systematically sampled operating conditions is required to establish these bounds and define the reliable application range of the model.

## Supplementary Material



## Data Availability

The code of this work can be found at https://github.com/SenyuanGu/Water-Feature-Selection.

## Notes

Wastewater treatment plant process scheme and bioreactor configuration (Section 1: Figures S1–S2); key biochemical and kinetic parameters of the ASM1-N_2_O model (Section 2: Table S1); feature engineering including state variable classification and model simulation results (Section 2: Tables S2–S3 and Figures S3–S5); hyperparameter tuning search space and optimal configurations (Section 3: Tables S4–S5); computational performance summary (Section 3: Table S6); feature selection methodology including mechanistic sensitivity analysis and SC-score derivation (Section 4); LLM prompt design for RAG-based feature recommendation (Section 5); model performance comparisons of pure machine learning and attention-based LSTM models (Section 6: Table S7); LASSO and SHAP analysis results (Section 7: Tables S8–S11); LLM-RAG feature selection reports from GPT, Claude, and Gemini (Section 8); and generalizability analysis (Section 9: Figure S6) (PDF)
