# Supplementary material for: Knowledge-Based Feature Selection Substantially Enhances Data-Driven Wastewater Treatment Modeling
Source: Environ Sci Technol. 2026 Jul 12;60(28):19882–94. doi: 10.1021/acs.est.6c04963 (PMC13394409; doi:10.1021/acs.est.6c04963)
Supplement: Supplementary file 1 [file es6c04963_si_001.pdf]

# Knowledge-based Feature Selection Substantially Enhances Data-driven Wastewater Treatment Modelling

Senyuan Gu [1,2], Shuting Wang [3], Ruihong Qiu [4], Kaili Li [3], Jaswinder Manjeet Singh [5], Jue Zhang [6], Bing-Jie Ni [1], T. David Waite [1,7], Liu Ye [3] and Haoran Duan [1,2,3,8] *

[1] UNSW Water Research Centre, School of Civil and Environmental Engineering, The University of New South Wales, Sydney, NSW 2052, Australia

[2] Australian Centre for Water and Environmental Biotechnology (ACWEB, formerly AWMC), The University of Queensland, Brisbane, QLD 4072, Australia

[3] School of Chemical Engineering, The University of Queensland, Brisbane, QLD 4072, Australia

[4] School of Information Technology and Electrical Engineering, The University of Queensland, Brisbane, QLD 4072, Australia

5 Gold Coast City Council, Southport, QLD 4215, Australia

[6] College of Geo-informatics, Zhejiang University of Technology, Zhejiang Province, P. R. China

[7] UNSW Centre for Transformational Environmental Technologies, Yixing, Jiangsu Province 214206, P. R. China

[8] Department of Civil Engineering, The University of Hong Kong, Pokfulam, Hong Kong SAR, China

*Corresponding Author: *haoran.duan@hku.hk*

The Supporting Information provides additional methodological details, supplementary figures and tables, and extended benchmarking results that support the main manuscript. Specifically, **Section 1** presents schematic diagrams of the two full-scale wastewater treatment plants studied; **Section 2** provides detailed feature-engineering information, including ASM-$N_2O$ model parameters, simulated state variables, and classification of reactor-state variables; **Section 3** summarizes hyperparameter search spaces, optimal model configurations, and computational resource requirements; **Section 4** describes the mathematical formulation of the mechanistic feature selection procedure; **Section 5** gives the LLM prompt used for LLM-RAG-assisted feature selection; **Section 6** reports supplementary benchmarking results for pure machine-learning and attention-based LSTM models, including statistical comparisons; **Section 7** presents LASSO- and SHAP-based feature selection results and their comparative performance; and **Section 8** provides representative LLM-RAG feature selection reports with supporting references and rationale.

**(49 pages, 6 figures, 13 tables)**

## Section 1: WWTP Process Scheme and Schematic Diagram

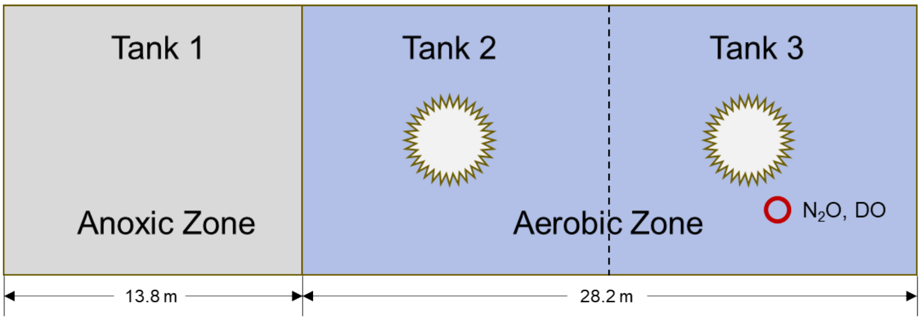


**Figure S1.** Schematic of the target bioreactor of WWTP-A.

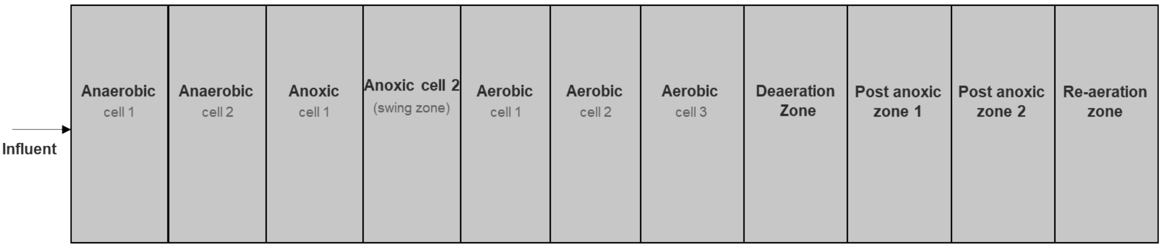


**Figure S2.** Schematic of the target bioreactor of WWTP-B.

## Section 2: Feature Engineering

**Table S1.** Key biochemical and kinetic parameters of the ASM1-$N_2O$ model.

| Name | Definition | Unit | CASE 1 | CASE 2 | Source |
|---|---|---|---|---|---|
| fXI | Fraction of inert COD in biomass | $g\ COD \cdot g\ COD^{-1}$ | 0.08 | 0.08 | Henze et al. [1] |
| iNSS | Nitrogen content of soluble organic substrate | $g\ N \cdot g\ COD^{-1}$ | 0.03 | 0.03 | Henze et al. [1] |
| iNXB | Nitrogen content of biomass | $g\ N \cdot g\ COD^{-1}$ | 0.07 | 0.07 | Mozumder et al. [2] |
| iNXI | Nitrogen content of particulate inerts | $g\ N \cdot g\ COD^{-1}$ | 0.07 | 0.07 | Mozumder et al. [2] |
| YAOB | Yield of AOB | $g\ COD \cdot g\ N^{-1}$ | 0.15 | 0.15 | Ni et al. [3] |
| YAOB,1 | Yield of AOB ($NH_2OH$) | $g\ COD \cdot g\ N^{-1}$ | 0.1125 | 0.1125 | Wan et al. [4] |
| YAOB,2 | Yield of AOB (NO) | $g\ COD \cdot g\ N^{-1}$ | 0.0375 | 0.0375 | Wan et al. [4] |
| YNOB | Yield of NOB | $g\ COD \cdot g\ N^{-1}$ | 0.057 | 0.057 | Wiesmann [5] |
| YH | Yield of heterotrophs | $g\ COD \cdot g\ N^{-1}$ | 0.6 | 0.6 | Hiatt and Grady [6] |
| μAOB | Growth rate of AOB | $d^{-1}$ | 0.84 | 2.631685 | Calibrated |
| bAOB | Decay rate of AOB | $d^{-1}$ | 0.068 | 0.12 | Mozumder et al. [2] |
| ηAOB,ND | ND reduction factor | - | 0.21 | 0.0001 | Calibrated |
| ηAOB,NN | NN reduction factor | - | 0.53 | 0.5 | Calibrated |
| KAOB,1,O | DO half-sat ($NH_4$ ox) | $g\ O_2 \cdot m^{-3}$ | 0.52 | 0.5 | Calibrated |
| KAOB,2,O | DO half-sat ($NO_2$ prod) | $g\ O_2 \cdot m^{-3}$ | 2.0 | 0.073 | Calibrated |
| KAOB,HAO,NO | NO half-sat | $g\ N \cdot m^{-3}$ | 0.004 | 0.0003 | Calibrated |
| KAOB,NO2 | $NO_2$ half-sat | $g\ N \cdot m^{-3}$ | 0.2 | 0.2 | Pocquet et al. [7] |
| KAOB,NH2OH | $NH_2OH$ half-sat | $g\ N \cdot m^{-3}$ | 0.1 | 0.01 | Pocquet et al. [7] |
| KAOB,I,O | DO inhibition ($N_2O$) | $g\ O_2 \cdot m^{-3}$ | 0.8 | 0.8 | Pocquet et al. [7] |
| KAOB,ND,O | DO effect (ND) | $g\ N \cdot m^{-3}$ | 0.5 | 0.5 | Pocquet et al. [7] |
| KAOB,NH | $NH_4$ half-sat | $g\ N \cdot m^{-3}$ | 1 | 0.5 | Calibrated |
| KAOB,NN,NO | NO half-sat (NN) | $g\ N \cdot m^{-3}$ | 0.008 | 0.008 | Pocquet et al. [7] |
| μNOB | Growth rate of NOB | $d^{-1}$ | 0.79 | 2.39966 | Hellinga et al. [8] |
| bNOB | Decay rate of NOB | $d^{-1}$ | 0.04 | 0.06 | Mozumder et al. [2] |
| KNOB,NH | NH half-sat | $g\ N \cdot m^{-3}$ | 0.02 | 0.02 | Mozumder et al. [2] |
| KNOB,NO2 | $NO_2$ half-sat | $g\ N \cdot m^{-3}$ | 0.06 | 0.06 | Calibrated |
| KNOB,O | DO half-sat | $g\ O_2 \cdot m^{-3}$ | 1.0 | 0.01 | Wiesmann [5] |

| Name | Definition | Unit | CASE 1 | CASE 2 | Source |
|---|---|---|---|---|---|
| μH | Growth rate heterotrophs | $d^{-1}$ | 11.8 | 6 | Hiatt and Grady [6] |
| bH | Decay rate heterotrophs | $d^{-1}$ | 0.75 | 0.406 | Hiatt and Grady [6] |
| KH,NH | NH half-sat | $g\ N\cdot m^{-3}$ | 0.02 | 0.02 | Mozumder et al. [2] |
| KH,O | DO half-sat | $g\ O_2\cdot m^{-3}$ | 1.77 | 0.1 | Calibrated |
| KH,S | COD half-sat | $g\ COD\cdot m^{-3}$ | 20 | 20 | Hiatt and Grady [6] |
| ηY | Yield reduction (anoxic) | - | 0.9 | 0.9 | Hiatt and Grady [6] |
| ηg2 | Anoxic factor ($NO_3$) | - | 0.20 | 0.28 | Calibrated |
| ηg3 | Anoxic factor ($NO_2$) | - | 0.0139 | 0.16 | Calibrated |
| ηg4 | Anoxic factor (NO) | - | 0.335 | 0.35 | Calibrated |
| ηg5 | Anoxic factor ($N_2O$) | - | 0.154 | 0.35 | Calibrated |

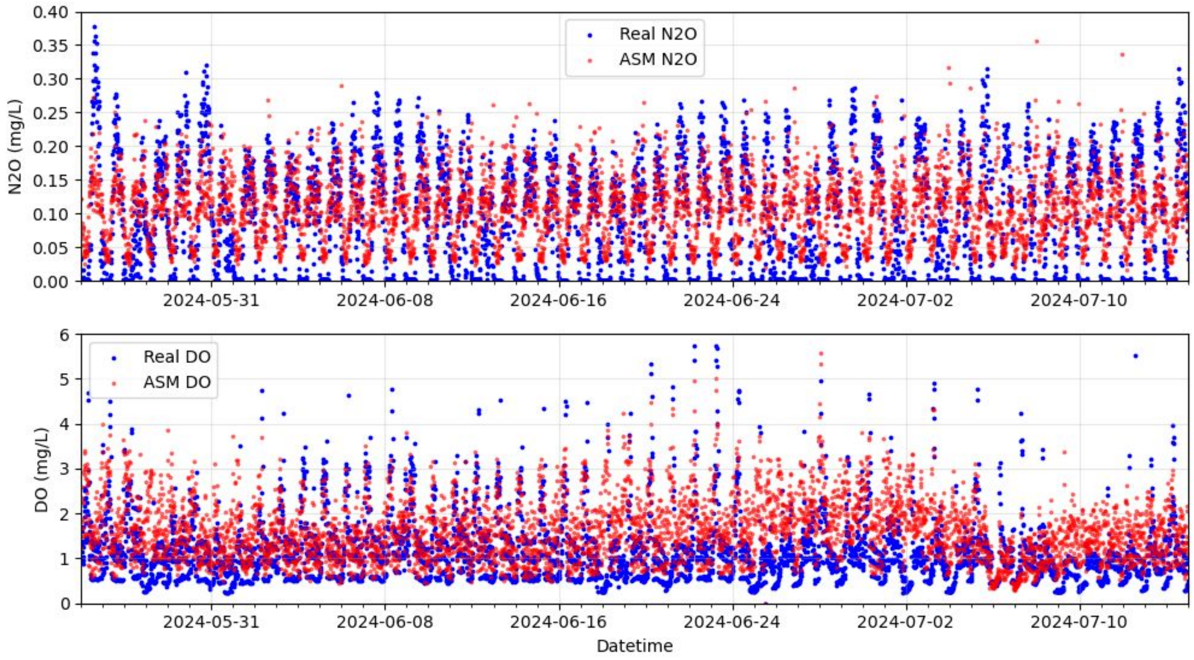


**Figure S3.** Time series of simulated state variables ($N_2O$, DO), MSE($N_2O$)=0.021 (WWTP-A).

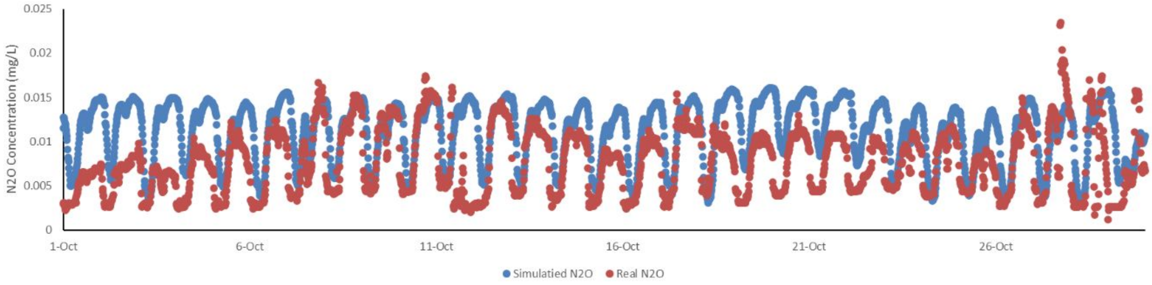

**Figure S4.** Time series of simulated N2O (WWTP-B).

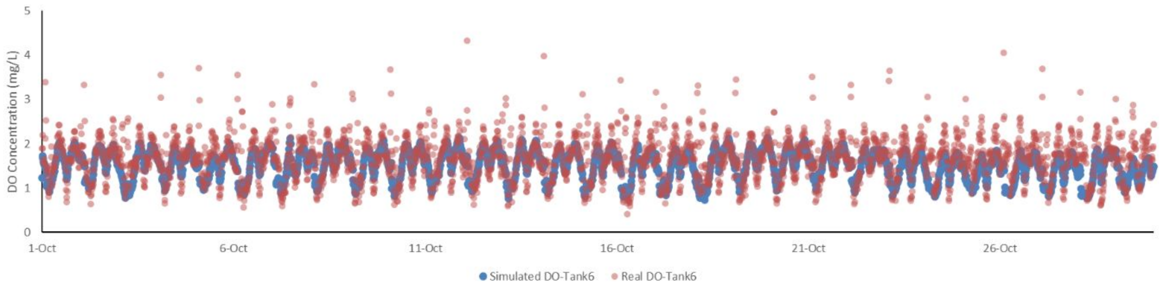


**Figure S5.** Time series of simulated DO (WWTP-B).

**Table S2.** Classification of state variables in the three reactor zones (tanks 1–3) (WWTP-A).

| | Tank1 | Tank2 | Tank3 |
|---|---|---|---|
| Nitrogen Species | $N_2$-Tank1 | $N_2$-Tank2 | $N_2$-Tank3 |
| | $N_2O$-Tank1 | $N_2O$-Tank2 | $N_2O$-Tank3 |
| | $NH_4^+$-Tank1 | $NH_4^+$-Tank2 | $NH_4^+$-Tank3 |
| | $NH_2OH$-Tank1 | $NH_2OH$-Tank2 | $NH_2OH$-Tank3 |
| | NO-Tank1 | NO-Tank2 | NO-Tank3 |
| | $NO_2^-$-Tank1 | $NO_2^-$-Tank2 | $NO_2^-$-Tank3 |
| | $NO_3^-$-Tank1 | $NO_3^-$-Tank2 | $NO_3^-$-Tank3 |
| DO | DO-Tank1 | DO-Tank2 | DO-Tank3 |
| COD | COD-Tank1 | COD-Tank2 | COD-Tank3 |
| Biomass | XAOB-Tank1 | XAOB-Tank2 | XAOB-Tank3 |
| | XH-Tank1 | XH-Tank2 | XH-Tank3 |
| | XI-Tank1 | XI-Tank2 | XI-Tank3 |
| | XNOB-Tank1 | XNOB-Tank2 | XNOB-Tank3 |

**Table S3.** Classification of state variables (WWTP-B).

| | Variables |
|---|---|
| Nitrogen Species | $N_2$, $N_2O$, $NH_4^+$, $NH_2OH$, NO, $NO_2^-$, $NO_3^-$ (Tank1–Tank12) |
| Oxygen & Substrate | DO, COD (Tank1–Tank12) |
| Biomass | X_AOB, X_H, X_I, X_NOB (Tank1–Tank12) |

**Section 3: Hyperparameter Tuning**

**Table S4.** Hyperparameter search space and optimal configurations

| Hyperparameter | Search space | LSTM | CNN-LSTM | FA-LSTM | LSTM-Attn |
|---|---|---|---|---|---|
| Architecture | | | | | |
| LSTM layers | {1,2,3,5} | — | 1 | 1 (fixed) | {1,2} |
| LSTM units | {32,64,128} | {32–128} | 64 | {32–128} | {32–128} |
| CNN filters | — | — | 32 | — | — |
| Dense units | {32,64,128} | — | 32 (fixed) | {32–128} | {32–128} |
| Attention heads | {2,4,8} | — | — | {2–8} | {2–8} |
| Dropout | [0.10–0.40] | — | CNN 0.21; LSTM 0.46; Dense 0.30 | multi-stage [0.10–0.40] | [0.10–0.50] |
| Attention dropout | [0.10–0.40] | — | — | [0.10–0.40] | [0.10–0.40] |
| L2 reg | [1e−5–1e−2] | — | 1.1e−5 | [1e−5–1e−3] | [1e−4–1e−2] |
| Optimiser | | | | | |
| Learning rate | [1e−5–2e−3] | [1e−5–1e−4] | 4.94e−4 | [1e−4–2e−3] | [1e−4–2e−3] |
| Huber δ | [0.01–0.5] | [0.05–0.5] | 0.0187 | [0.01–0.20] | [0.01–0.20] |
| Batch size | {64,128,256} | 128 | {64–256} | {64–256} | {64–256} |
| Training | | | | | |

Walk-forward CV (3 folds, 15% test size). Optuna minimizes weighted physical-space MAE. Fixed values are from the selected best configuration.

**Table S5.** Optimal hyperparameter configurations identified for each model variant.

| Hyperparameter | LSTM (§3.1) | LSTM (§3.2) | FA-LSTM (§3.1) | LSTM-Attn (§3.1) | LSTM-Attn (§3.4) | FA-LSTM (§3.4) |
|---|---|---|---|---|---|---|
| LSTM layers | 5 | 1 | 1 (fixed) | 2 | 1 | 1 (fixed) |
| LSTM units (layer 1) | 256 | 128 | 128 | 128 | 64 | 128 |
| LSTM units (layer 2) | 256 | — | — | 64 | — | — |
| LSTM units (layers 3–5) | 64 / 256 / 128 | — | — | — | — | — |
| Dense units | — | — | 128 | 128 | 64 | 128 |

| Hyperparameter | LSTM (§3.1) | LSTM (§3.2) | FA-LSTM (§3.1) | LSTM-Attn (§3.1) | LSTM-Attn (§3.4) | FA-LSTM (§3.4) |
|---|---|---|---|---|---|---|
| Attention heads | — | — | 8 | 2 | 4 | 2 |
| Batch normalisation | False | False | False | False | False | False |
| LSTM dropout | 0.10 | 0.25 | 0.25 | 0.35 / 0.40 | 0.20 / 0.50 | 0.25 |
| Feature / attention dropout | — | — | feat: 0.20; attn: 0.35 | attn: 0.25 | attn: 0.10 | feat: 0.10; attn: 0.30 |
| Fusion / dense dropout | — | — | 0.45 | 0.30 | 0.50 | 0.50 |
| L2 regularisation | — | — | $5.63\times10^{-4}$ | $2.59\times10^{-3}$ | $3.09\times10^{-3}$ | $1.60\times10^{-3}$ |
| Learning rate | $3.48\times10^{-3}$ | $1.92\times10^{-3}$ | $1.49\times10^{-3}$ | $1.56\times10^{-3}$ | $1.37\times10^{-3}$ | $1.56\times10^{-3}$ |
| Gradient clip norm | — | — | 0.233 | 0.372 | 0.402 | 0.342 |
| Huber δ | 0.065 | 0.021 | 0.019 | 0.161 | 0.031 | 0.030 |
| Batch size | 32 | 32 | 256 | 256 | 256 | 256 |

**Table S6.** Computational performance summary of the proposed framework.

| Component | Task | Hardware | Time (approx.) | Resource Usage |
|---|---|---|---|---|
| Expert-guided feature selection | SC-score computation | CPU | ~10 s | ~250 MB RAM |
| SHAP Computation | 52 features | GPU* | ~1 min | ~2.0 GB VRAM |
| LLM-RAG Pipeline | 15-round querying (3 LLMs) | API (cloud) | ~10 min total | ~30 k tokens per round |
| Hyperparameter Search | 100 Optuna trials | GPU* | ~2 h/model | ~2.5 GB VRAM |
| Model Training | FS-LSTM (20 runs) | GPU* | ~20 min | ~1.5 GB VRAM |
| Model Training | Attention-LSTM (20 runs) | GPU* | ~25 min | ~2.5 GB VRAM |

*Hardware resources were allocated dynamically by the HPC scheduler (PBS); GPU assignments varied across jobs.

**Section 4: Feature Selection**

(1) Mass balance of $N_2O$ at pseudo-steady state

For each continuous-flow reactor r (r=1,2,3r = 1,2,3r=1,2,3), the overall mass balance of $N_2O$ is

$$\frac{dC_{N_2O,r}}{dt} = \frac{Q}{V_r}\left(C_{N_2O,in} - C_{N_2O,r}\right) + \sum_{k=1}^{N_{rxn}} r_{k,r}(X,\theta)$$

where Q is the flow rate, Vr is the reactor volume, $r_{k,r}$ is the rate of the $k^{th}$ mechanistic reaction (AOB, NOB, heterotrophic, etc.), X is the vector of state variables (substrates, biomass, DO, …), and θ is the kinetic-parameter vector.

At pseudo-steady state (dC/dt=0),

$$0 = \frac{Q}{V_r}\left(C_{N_2O,in} - C^*_{N_2O,r}\right) + \sum_{k} r_{k,r}\,!(X^*,\theta)$$

giving the baseline steady-state concentration $C^*_{N_2O,r}$.

(2) Single-factor perturbation

For any candidate input/state variable $x_i$ (either an influent component or an initial state), apply a relative perturbation

$$x_i' = x_i^*!(1+\delta), \qquad \delta = +5\%$$

Note that the final mutual information (MI) scores are normalized; therefore, the choice of perturbation magnitude (e.g., within 5–15%) does not significantly affect the relative MI rankings.

Solving Eq. (2) again with the perturbed $x_i'$ yields a new steady concentration

$$C'_{N_2O,r} = C^*_{N_2O,r} + \Delta C_{N_2O,r}$$

where

$$\Delta C_{N_2O,r} = C'_{N_2O,r} - C^*_{N_2O,r}, \qquad \Delta x_i = x_i' - x_i^*$$

(3) Dimensionless mechanistic sensitivity coefficient

Using a first-order Taylor expansion,

$$\Delta C_{N_2O,r} \approx \frac{\partial C_{N_2O,r}}{\partial x_i} \Delta x_i$$

Define the Mechanistic Sensitivity Coefficient (MSC) as

$$S_i = \frac{\frac{\Delta C_{N_2O,r}}{C^*_{N_2O,r}}}{x_i/x^*_i} = \frac{\partial \ln C_{N_2O,r}}{\partial \ln x_i}$$

(4) Normalised Mechanistic Importance (MI) score

To compare variables on a common scale, we normalise the absolute $S_i$ values:

$$\mathrm{MI_i} = \sqrt{\frac{|S_j|}{\max_j |S_j|}}, \quad 0 \le \mathrm{MI_i} \le 1$$

For variables obtained from online sensors, the $\mathrm{MI_i}$ values were manually assigned to reflect measurement reliability.

$\mathrm{MI_i}$ is the weight used in the *Kinetic Equation-Based Feature Weighting* step and feeds directly into feature selection and model interpretation.

**Section 5: LLM Prompt**

You are an expert in wastewater treatment process modeling. You are tasked with simulating $N_2O$ emissions in the biological treatment section of a wastewater treatment plant. The system includes one anaerobic tank followed by two aerobic tanks. Your focus is on modeling the liquid-phase $N_2O$ emissions in the third tank (i.e., the second aerobic tank) using an LSTM-based model.

I will provide a dataset containing a wide range of process features as input.

Do NOT create new features, rename features, or select variables that are not explicitly listed in the table.

Your job is to identify the best 7 features for the LSTM model to achieve the best possible performance.

Please perform the following steps:

1. Analyze the dataset I provide (includes time-series measurements of various process variables).

2. Evaluate all available features ONLY from the provided feature table.

3. Evaluate these features from multiple perspectives such as:

- Relevance to $N_2O$ production pathways

- Temporal predictive value for LSTM

- Process interpretability

- Redundancy with other variables

4. Rate each feature under these perspectives on a scale from 1 (low) to 5 (high), and organize the results into a feature rating matrix.

5. Based on the evaluation, select exactly 7 features ONLY from the provided table.

6. Generate a report summarizing:

- The selected 7 features

- The reasons each was chosen

- Any additional notes on how these features are expected to improve model performance.

Remember: the final selected features must come strictly from the provided feature table.

**Section 6: Model Performance of Pure Machine Learning models and attention based LSTM models**

**Table S7.** Prediction performance of LSTM models with different depths and attention mechanisms.

| Model 1 | Model 2 | Metric | Mean (FS) | Mean (Full) | p (adj.) | |
|---|---|---|---|---|---|---|
| Basic-LSTM (FS) | Baseline | MAE | 0.0367 | 0.0665 | <0.001 | *** |
| | LSTM-5L | | 0.0367 | 0.0567 | <0.001 | *** |
| | LSTM-Attn | | 0.0367 | 0.0478 | <0.001 | *** |
| | Attn-LSTM | | 0.0367 | 0.0326 | 0.0067 | * |
| | Baseline | $R^2$ | 0.692 | 0.122 | <0.001 | *** |
| | LSTM-5L | | 0.692 | 0.226 | <0.001 | *** |
| | LSTM-Attn | | 0.692 | 0.465 | <0.001 | *** |
| | Attn-LSTM | | 0.692 | 0.712 | 0.9705 | ns |
| Tuned-LSTM(FS) | Baseline | MAE | 0.0330 | 0.0665 | <0.001 | *** |
| | LSTM-5L | | 0.0330 | 0.0567 | <0.001 | *** |
| | LSTM-Attn | | 0.0330 | 0.0478 | <0.001 | *** |
| | Attn-LSTM | | 0.0330 | 0.0326 | 1.000 | ns |
| | Baseline | $R^2$ | 0.722 | 0.122 | <0.001 | *** |
| | LSTM-5L | | 0.722 | 0.226 | <0.001 | *** |
| | LSTM-Attn | | 0.722 | 0.465 | <0.001 | *** |
| | Attn-LSTM | | 0.722 | 0.712 | 1.000 | ns |

Mann–Whitney U test (two-sided), n = 20 runs per model. * $p < 0.05$, ** $p < 0.01$, *** $p < 0.001$.

**Table S8.** LSTM architecture configurations used for the model-depth sensitivity analysis.

| Architecture | LSTM units per layer | Dropout rate after each LSTM layer | Trainable parameters |
|---|---|---|---|
| 1-layer LSTM | 32 | 0.2 | 7,073 |
| 2-layer LSTM | 32 / 32 | 0.2 | 15,521 |
| 3-layer LSTM | 32 / 32 / 32 | 0.2 | 23,969 |
| 5-layer LSTM | 64 / 64 / 128 / 64 / 64 | 0.3 | 237,889 |
| 7-layer LSTM | 64 / 64 / 64 / 128 / 64 / 64 / 64 | 0.3 | 304,449 |

All architectures used the same seven expert-selected features. Batch normalization was applied after each LSTM layer. All models used a 96-step input sequence corresponding to a 24 h look-back window at 15 min resolution, a linear single-unit output layer, the Adam

optimizer with an initial learning rate of $1 \times 10^{-3}$, Huber loss, a batch size of 64, and a maximum of 150 epochs. Each architecture was independently trained 20 times using different random initializations.

**Table S9.** LSTM architecture configurations used for the model-depth sensitivity analysis.

| LSTM configuration | Runs | Test MAE, mean ± SD | Test $R^2$, mean ± SD |
|---|---|---|---|
| 1-layer LSTM | 20 | 0.0413 ± 0.0027 | 0.6536 ± 0.0429 |
| 2-layer LSTM (FS-Basic-LSTM) | 20 | 0.0367 ± 0.0034 | 0.6919 ± 0.0449 |
| 3-layer LSTM | 20 | 0.0364 ± 0.0042 | 0.6876 ± 0.0624 |
| 5-layer LSTM | 20 | 0.0353 ± 0.0051 | 0.6955 ± 0.0769 |
| 7-layer LSTM | 20 | 0.0404 ± 0.0068 | 0.6154 ± 0.1195 |
| FS-tuned-LSTM (1 layer) | 20 | 0.0330 ± 0.0014 | 0.7225 ± 0.0263 |

The architectural configurations of the predefined LSTM models are provided in Table S9, while the hyperparameters of the FS-tuned-LSTM are reported in Table S5.

**Section 7: LASSO and SHAP Analysis Results**

**Benchmark feature selection methods**

To benchmark the proposed knowledge-based feature selection framework, LASSO regularization and SHAP-based feature importance analysis were independently applied. For LASSO analysis, the input variables were standardized and evaluated through 100 repeated subsampling runs, each using 80% of the available training data. The regularization parameter was determined using five-fold cross-validation, and features with non-zero coefficients were recorded. Features selected in at least 80% of the runs were considered stable.

For the SHAP-based analysis, a GradientExplainer was applied to the trained five-layer LSTM model. SHAP values were aggregated across timesteps and derived rolling features to quantify the overall contribution of each original input variable. Features were ranked according to their mean absolute SHAP values, and the seven highest-ranked features were selected to ensure a controlled comparison with the proposed method. Further details and results are provided in the Supporting Information.

**Table S10**: LASSO analysis results.

| Feature | Coefficient |
|---|---|
| S3N2 | -2.97e-02 |
| S1NH2OH | 2.54e-02 |
| S3NO | 1.92e-02 |
| S1S | 1.62e-02 |
| S2N2O | 1.52e-02 |
| S2NH | 1.44e-02 |
| S1NO2 | -1.14e-02 |
| S3NH2OH | 1.03e-02 |

| Feature | Coefficient |
|---|---|
| X1H | -8.92e-03 |
| S3NH | 7.87e-03 |
| Aeration Power2 | -6.03e-03 |
| X3I | -5.82e-03 |
| S3O | 5.72e-03 |
| X2I | -4.94e-03 |
| X1NOB | 4.10e-03 |
| Aeration Power1 | -3.80e-03 |
| Effluent NITRATE | 3.72e-03 |
| X1AOB | 3.10e-03 |
| Ammonium | 2.82e-03 |
| InflowRate | 1.90e-03 |
| DO | -1.03e-03 |
| WAS | 2.11e-07 |

**Table S11.** LASSO selected feature results.

| Model 1 | Model 2 | Metric | Mean (LASSO) | Mean (Other) | p (adj.) | |
|---|---|---|---|---|---|---|
| LASSO-selected tuned LSTM | LSTM-5-Layers | MAE | 0.064 | 0.057 | 0.048 | * |
| | | $R^2$ | 0.091 | 0.226 | 0.090 | ns |
| | FS-basic-LSTM | MAE | 0.064 | 0.037 | <0.001 | *** |
| | | $R^2$ | 0.091 | 0.692 | <0.001 | *** |
| | FS-tuned-LSTM | MAE | 0.064 | 0.033 | <0.001 | *** |
| | | $R^2$ | 0.091 | 0.723 | <0.001 | *** |

**Table S12.** SHAP analysis results.

| **Feature** | **Mean Absolute SHAP Value** | **Mean SHAP Value** | **Standard Deviation of SHAP** |
|---|---|---|---|
| WAS | 0.000742466 | -4.37E-05 | 0.000816 |
| Rainfall | 0.000198548 | -0.00015 | 0.000479 |
| Aeration Power2 | 0.000195297 | 2.24E-05 | 0.000205 |
| $NH_4^+$-Tank1 | 0.000174811 | 7.22E-05 | 0.000216 |
| RAS_CDE | 0.000174394 | 5.88E-05 | 0.000185 |
| RAS_F | 0.000174211 | 6.03E-05 | 0.000188 |
| Nitrate | 0.000169108 | -0.00017 | 0.000148 |

**Table S13.** Pairwise statistical comparison of the SHAP-selected tuned LSTM against alternative models across 20 independent runs.

| Model 1 | Model 2 | Metric | Mean (SHAP) | Mean (Other) | p (adj.) | |
|---|---|---|---|---|---|---|
| SHAP-selected Tuned LSTM | LSTM-5-Layers | MAE | 0.0407 | 0.0567 | <0.001 | *** |
| | | $R^2$ | 0.5975 | 0.2263 | <0.001 | *** |
| | FS-basic-LSTM | MAE | 0.0407 | 0.0367 | 0.0015 | ** |
| | | $R^2$ | 0.5975 | 0.6919 | <0.001 | *** |
| | FS-tuned-LSTM | MAE | 0.0407 | 0.0330 | <0.001 | *** |
| | | $R^2$ | 0.5975 | 0.7225 | <0.001 | *** |

Mann–Whitney U test (two-sided), n = 20 runs per model. * $p < 0.05$, ** $p < 0.01$, *** $p < 0.001$.

The results show that the SHAP-selected tuned LSTM significantly outperforms the full-feature LSTM model (LSTM-5-Layers), achieving lower MAE (0.0407 vs 0.0567) and higher $R^2$ (0.5975 vs 0.2263), with both differences being highly significant ($p < 0.001$). However, when compared with feature selection-based models (FS-basic-LSTM and FS-tuned-LSTM), the SHAP-selected tuned LSTM demonstrates inferior performance. Specifically, it has significantly higher MAE than both FS-basic-LSTM (0.0407 vs 0.0367, $p = 0.0015$) and FS-tuned-LSTM (0.0407 vs 0.0330, $p < 0.001$), as well as significantly lower $R^2$ values (0.5975 vs 0.6919 and 0.7225, respectively; both $p < 0.001$).

**Section 8:** LLM- RAG feature selection report

**GPT-Report 1**

**1–2. Dataset-driven feature analysis and evaluation perspectives**

I evaluated all candidate predictors under four perspectives: **P1 mechanistic relevance** (known drivers of $N_2O$ formation/stripping in aerobic systems), **P2 statistical signal** (|corr($N_2O$)| in the provided matrix), **P3 non-redundancy** (avoids duplicating the same information across highly collinear mechanistic-state variables), and **P4 operational availability/actionability** (measured/manipulated variables preferred to internal simulated states, unless they add unique predictive content). The choice of DO, nitrogen species, temperature/flow, and aeration proxies is consistent with prior full-scale LSTM/DNN studies of $N_2O$ dynamics. (Kim2020 lines 0-16)

**3. Feature rating matrix (1=low, 5=high)**

| Feature | P1 | P2 | P3 | P4 |
|---|---|---|---|---|
| S2N2O | 5 | 5 | 2 | 2 |
| S3N2O | 5 | 5 | 2 | 2 |
| S2NO | 5 | 5 | 3 | 2 |
| S3NO | 5 | 5 | 3 | 2 |
| S1NH | 4 | 5 | 2 | 2 |
| S3NO3 | 4 | 4 | 3 | 2 |
| S3O | 5 | 4 | 3 | 3 |
| DO | 5 | 4 | 4 | 5 |
| InflowRate | 4 | 3 | 5 | 5 |
| Aeration Power2 | 4 | 3 | 4 | 5 |
| Aeration On/Off | 4 | 3 | 4 | 5 |
| Ammonium | 4 | 1 | 4 | 5 |
| Nitrate | 4 | 1 | 4 | 5 |

**4. Selected exactly 7 features (for Tank 3 liquid-phase $N_2O$ LSTM)**

**DO; Aeration Power2; Aeration On/Off; InflowRate; S3N2O; S3NO; S3NO3.**

**5. Report (rationale and expected impact)**

**DO** captures aerobic redox control affecting nitrification/denitrification pathways and correlates negatively with $N_2O$ here. (Kim2020 lines 0-16)

**Aeration Power2** and **Aeration On/Off** provide manipulated forcing on oxygen transfer and stripping dynamics not fully represented by DO alone. (Kim2020 lines 0-16)

**InflowRate** encodes hydraulic/load disturbances repeatedly identified as influential for $N_2O$ variability. (Kim2020 lines 0-16)

**S3N2O** supplies a high-signal latent state (strong correlation) aligned with the target tank, improving short-horizon forecast skill. (Kim2020 lines 0-16)

**S3NO** (NO) and **S3NO3** (nitrate) represent key nitrogen-oxide intermediates/products shaping $N_2O$ production potential, complementing DO/aeration drivers while reducing reliance on weakly correlated bulk effluent $NH_4^+/NO_3^-$ signals. (Kim2020 lines 0-16)

Additional note: the inclusion of mechanistic-state proxies (S3*) follows the "hybrid" direction suggested for improving deep-learning $N_2O$ models when critical internal variables are otherwise unmeasured. (An2021 lines 20-30)

References


1. (Kim2020 lines 0-16): Kim, J., et al. "Integrated Model for Understanding N2O Emissions from Wastewater Treatment Plants: A Deep Learning Approach." *Environmental Science & Technology*, vol. 54, no. 24, 2020, pp. 15716-15725. DOI: 10.1021/acs.est.0c05231.
2. (An2021 lines 20-30): "An Integrated First Principal and Deep Learning Approach for Modeling Nitrous Oxide Emissions from Wastewater Treatment Plants." *Environmental Science & Technology*, vol. 55, no. 21, 2021, pp. 14450-14460. DOI: 10.1021/acs.est.1c05020.


**GPT-Report 2**

**Feature rating matrix (1=low, 5=high)**

Scoring perspectives: (i) relevance to established $N_2O$ pathways (AOB nitrification / nitrifier denitrification under low DO; dependence on $NH_4^+$/NOx/DO and historical $N_2O$), (ii) temporal predictive value for LSTM, (iii) process interpretability, (iv) redundancy risk (higher score = *lower* redundancy). (Pan2024 lines 0-18, Pan2024 lines 107-122, Kim2020 lines 16-30)

| Feature (from table) | Pathway relevance | LSTM temporal value | Interpretability | Low redundancy |
|---|---|---|---|---|
| DO | 5 | 4 | 5 | 4 |
| InflowRate | 4 | 4 | 5 | 4 |
| Ammonium | 4 | 3 | 5 | 3 |
| Nitrate | 3 | 3 | 5 | 3 |
| Aeration Power2 | 4 | 4 | 4 | 3 |
| Aeration On/Off | 4 | 4 | 4 | 3 |
| S3N2O | 5 | 5 | 4 | 5 |

| Feature (from table) | Pathway relevance | LSTM temporal value | Interpretability | Low redundancy |
| --- | --- | --- | --- | --- |
| S3NH | 5 | 4 | 4 | 4 |
| S3NO | 5 | 4 | 4 | 4 |
| S3NO2 | 5 | 4 | 4 | 4 |
| S3O | 4 | 3 | 4 | 3 |

**Selected 7 features (exactly)**

1) **S3N2O** — strongest direct dynamic proxy for tank-3 $N_2O$ formation/accumulation; complements LSTM sequence learning. (An2021 lines 30-42, Pan2024 lines 107-122)
2) **DO** — primary control variable for nitrifier denitrification and incomplete oxidation regimes driving $N_2O$. (Pan2024 lines 0-18, Kim2020 lines 16-30)
3) **S3NH** — captures $NH_4^+$ availability for AOB activity and associated $N_2O$ pathways in the modeled location. (Pan2024 lines 0-18, Pan2024 lines 107-122)
4) **S3NO** — represents NOx pool dynamics relevant to nitrifier/heterotrophic denitrification contributions to $N_2O$. (Pan2024 lines 0-18, Pan2024 lines 107-122)
5) **S3NO2** — nitrite is a key intermediate linked to AOB-related $N_2O$ peaks under low DO. (Pan2024 lines 0-18, Pan2024 lines 107-122)
6) **InflowRate** — major disturbance variable repeatedly identified as influential for $N_2O$ forecasting. (Pan2024 lines 0-18, Pan2024 lines 107-122, Kim2020 lines 16-30)
7) **Aeration Power2** — actuator/forcing for oxygen transfer and intermittency effects; adds operational context beyond DO alone. (Kim2020 lines 16-30)

**Notes on expected performance impact**

This set prioritizes mechanistically relevant drivers (DO, $NH_4^+$, NOx) plus an operational forcing (aeration) and a high-signal mechanistic proxy at tank-3 (S3N2O), aligning with prior findings that past/nearby $N_2O$ dynamics and flow/DO/N-species dominate LSTM forecast skill and interpretability. (Pan2024 lines 0-18, Pan2024 lines 107-122, Kim2020 lines 16-30, An2021 lines 30-42)

References


1. (Pan2024 lines 0-18): Pan, Y., Seshan, H., Massara, T. M., Daelman, M. R. J., Gruber, W., Kosonen, H., Guo, L., Vanrolleghem, P. A., & Mampaey, K. "Forecasting Nitrous Oxide Emissions from a Full-Scale Wastewater Treatment Plant Using LSTM-Based Deep Learning Models." *Water Research*, vol. 2024, 2024, p. 122754. doi:10.1016/j.watres.2024.122754.
2. (Pan2024 lines 107-122): Pan, Y., Seshan, H., Massara, T. M., Daelman, M. R. J., Gruber, W., Kosonen, H., Guo, L., Vanrolleghem, P. A., & Mampaey, K. "Forecasting

Nitrous Oxide Emissions from a Full-Scale Wastewater Treatment Plant Using LSTM-Based Deep Learning Models." *Water Research*, vol. 2024, 2024, p. 122754. doi:10.1016/j.watres.2024.122754.

3. (Kim2020 lines 16-30): Kim, J., et al. "Integrated Model for Understanding N2O Emissions from Wastewater Treatment Plants: A Deep Learning Approach." *Environmental Science & Technology*, vol. 54, no. 24, 2020, pp. 15716-15725. DOI: 10.1021/acs.est.0c05231.

4. (An2021 lines 30-42): "An Integrated First Principal and Deep Learning Approach for Modeling Nitrous Oxide Emissions from Wastewater Treatment Plants." *Environmental Science & Technology*, vol. 55, no. 21, 2021, pp. 14450-14460. DOI: 10.1021/acs.est.1c05020.

**GPT-Report3**

**Feature rating matrix (1=low, 5=high)**

| Feature (from table) | Relevance to $N_2O$ pathways | Temporal value for LSTM | Interpretability | Non-redundancy |
|---|---|---|---|---|
| DO | 5 | 5 | 5 | 4 |
| InflowRate | 4 | 4 | 5 | 4 |
| Aeration Power2 | 4 | 4 | 4 | 3 |
| Aeration On/Off | 4 | 4 | 4 | 3 |
| S3NH | 5 | 4 | 4 | 3 |
| S3NO2 | 5 | 4 | 4 | 3 |
| S3NO3 | 4 | 4 | 4 | 3 |
| S3O | 4 | 4 | 4 | 2 |
| S3N2O | 5 | 4 | 3 | 1 |
| S2N2O | 4 | 3 | 3 | 2 |
| Ammonium | 3 | 3 | 4 | 2 |
| Nitrate | 2 | 2 | 4 | 2 |

Ratings prioritize variables aligned with prior deep-learning $N_2O$ studies (DO, nitrogen species, temperature/flow/airflow) and hybrid mechanistic+LSTM inputs (simulated $NH_4$/$NO_2$/$NO_3$/DO) under limited observability. (Kim2020 lines 16-30, An2021 lines 30-42, An2021 lines 20-30)

**Selected 7 features (exactly)**

1) **DO** — primary control on nitrification/denitrification switching and a core predictor in prior LSTM/DNN $N_2O$ models. (Kim2020 lines 16-30)

2) **InflowRate** — captures load/hydraulic transients driving N transformations and $N_2O$ dynamics; used as a key DL input. (Kim2020 lines 16-30)

3) **Aeration Power2** — proxy for oxygen transfer/stripping intensity in the second aerobic tank, complementing DO with actuation information. (Kim2020 lines 16-30)

4) **Aeration On/Off** — encodes intermittency and regime shifts that an LSTM can exploit for delayed $N_2O$ responses. (Kim2020 lines 16-30)

5) **S3NH** — mechanistic surrogate for ammonium in tank 3, consistent with hybrid workflows using simulated $NH_4$ as DL input. (An2021 lines 30-42)

6) **S3NO2** — nitrite is a direct precursor/indicator of pathways associated with $N_2O$ peaks; mechanistic profiles are commonly fed to LSTM. (An2021 lines 30-42)

7) **S3NO3** — captures oxidized N pool and denitrification potential; included in mechanistic-to-LSTM feature sets. (An2021 lines 30-42)

**Notes**

I exclude **S3N2O/S2N2O** despite high correlation because they risk leakage/over-reliance on a mechanistic $N_2O$ proxy rather than learning from causal drivers; hybrid approaches in the cited work used mechanistic states excluding $N_2O$ as inputs to DL. (An2021 lines 30-42)

References


1. (Kim2020 lines 16-30): Kim, J., et al. "Integrated Model for Understanding N2O Emissions from Wastewater Treatment Plants: A Deep Learning Approach." *Environmental Science & Technology*, vol. 54, no. 24, 2020, pp. 15716-15725. DOI: 10.1021/acs.est.0c05231.

2. (An2021 lines 30-42): "An Integrated First Principal and Deep Learning Approach for Modeling Nitrous Oxide Emissions from Wastewater Treatment Plants." *Environmental Science & Technology*, vol. 55, no. 21, 2021, pp. 14450-14460. DOI: 10.1021/acs.est.1c05020.

3. (An2021 lines 20-30): "An Integrated First Principal and Deep Learning Approach for Modeling Nitrous Oxide Emissions from Wastewater Treatment Plants." *Environmental Science & Technology*, vol. 55, no. 21, 2021, pp. 14450-14460. DOI: 10.1021/acs.est.1c05020.


**GPT-Report4**

**1–4) Feature rating matrix (1=low, 5=high)**

Definitions: **AOB** = ammonia-oxidizing bacteria; **NOx** = $NO_2^-$+$NO_3^-$ (oxidized nitrogen). (Pan2024 lines 107-122, Pan2024 lines 0-18)

| Feature (from your table) | Pathway relevance | Temporal value for LSTM | Interpretability | Non-redundancy |
|---|---|---|---|---|
| DO | 5 | 5 | 5 | 4 |
| InflowRate | 4 | 4 | 5 | 4 |
| Ammonium | 5 | 4 | 5 | 3 |
| Nitrate | 3 | 3 | 4 | 3 |
| Aeration Power2 | 4 | 4 | 4 | 3 |
| Aeration On/Off | 4 | 4 | 4 | 3 |
| S3N2O | 5 | 5 | 3 | 2 |
| S3NH | 4 | 4 | 3 | 2 |
| S3NO | 4 | 4 | 3 | 2 |
| S3NO2 | 4 | 4 | 3 | 2 |
| S3NO3 | 3 | 3 | 3 | 2 |

Process-variable prioritization (DO, $NH_4^+$/NOx, inflow, aeration) follows full-scale LSTM feature-importance findings for $N_2O$ forecasting. (Pan2024 lines 107-122, Pan2024 lines 0-18) Candidate inputs used successfully in prior DNN/LSTM WWTP studies also include influent flow, air/aeration, temperature, $NH_4^+$, $NO_3^-$, DO, and historical $N_2O$. (Kim2020 lines 0-16, Kim2020 lines 16-30)

**5) Selected best 7 features (exactly 7; all from your table)**

**DO; InflowRate; Ammonium; Nitrate; Aeration Power2; Aeration On/Off; S3N2O.** (Kim2020 lines 0-16, Pan2024 lines 107-122, Kim2020 lines 16-30, Pan2024 lines 0-18)

**6) Report (rationale and expected impact)**

**DO** captures aerobic control on nitrification-linked $N_2O$ pathways and is repeatedly identified as a key predictor. (Pan2024 lines 107-122, Pan2024 lines 0-18)

**InflowRate** proxies load/hydraulics and was a top driver after historical $N_2O$ in encoder–decoder LSTM models. (Pan2024 lines 107-122, Pan2024 lines 0-18)

**Ammonium** represents AOB substrate and is consistently important for $N_2O$ forecasting. (Pan2024 lines 107-122, Pan2024 lines 0-18)

**Nitrate** provides oxidized-nitrogen state information (NOx proxy) used in prior deep models for $N_2O$. (Kim2020 lines 0-16, An2021 lines 20-30)

**Aeration Power2** and **Aeration On/Off** represent oxygen transfer forcing in the modeled tank, aligning with prior use of airflow/aeration as $N_2O$ predictors. (Kim2020 lines 0-16, Kim2020 lines 16-30)

**S3N2O** functions as a high-signal, tank-specific dynamic surrogate for local $N_2O$ state, providing strong short-horizon memory analogous to "past $N_2O$" being the dominant LSTM input. (Pan2024 lines 107-122, Pan2024 lines 0-18)

Redundancy note: mechanistic S3 variables (S3NH/S3NOx) are excluded because they are collinear with measured $NH_4^+/NO_3^-$ and DO, while S3N2O uniquely supplies direct $N_2O$-state information. (Pan2024 lines 107-122, Pan2024 lines 0-18)

References


1. (Pan2024 lines 107-122): Pan, Y., Seshan, H., Massara, T. M., Daelman, M. R. J., Gruber, W., Kosonen, H., Guo, L., Vanrolleghem, P. A., & Mampaey, K. "Forecasting Nitrous Oxide Emissions from a Full-Scale Wastewater Treatment Plant Using LSTM-Based Deep Learning Models." *Water Research*, vol. 2024, 2024, p. 122754. doi:10.1016/j.watres.2024.122754.
2. (Pan2024 lines 0-18): Pan, Y., Seshan, H., Massara, T. M., Daelman, M. R. J., Gruber, W., Kosonen, H., Guo, L., Vanrolleghem, P. A., & Mampaey, K. "Forecasting Nitrous Oxide Emissions from a Full-Scale Wastewater Treatment Plant Using LSTM-Based Deep Learning Models." *Water Research*, vol. 2024, 2024, p. 122754. doi:10.1016/j.watres.2024.122754.
3. (Kim2020 lines 0-16): Kim, J., et al. "Integrated Model for Understanding N2O Emissions from Wastewater Treatment Plants: A Deep Learning Approach." *Environmental Science & Technology*, vol. 54, no. 24, 2020, pp. 15716-15725. DOI: 10.1021/acs.est.0c05231.
4. (Kim2020 lines 16-30): Kim, J., et al. "Integrated Model for Understanding N2O Emissions from Wastewater Treatment Plants: A Deep Learning Approach." *Environmental Science & Technology*, vol. 54, no. 24, 2020, pp. 15716-15725. DOI: 10.1021/acs.est.0c05231.
5. (An2021 lines 20-30): "An Integrated First Principal and Deep Learning Approach for Modeling Nitrous Oxide Emissions from Wastewater Treatment Plants." *Environmental Science & Technology*, vol. 55, no. 21, 2021, pp. 14450-14460. DOI: 10.1021/acs.est.1c05020.


**GPT-Report 5**

**Feature rating matrix (1 = low, 5 = high)**

Scores reflect (i) relevance to $N_2O$ pathways, (ii) temporal predictive value for LSTM, (iii) process interpretability, and (iv) non-redundancy. (Pan2024 lines 0-18, Pan2024 lines 107-122, Pan2024 lines 55-72)

| Feature (from provided table) | Pathway relevance | Temporal predictive value | Interpretability | Non-redundancy |
|---|---|---|---|---|
| S3N2O (mechanistic sim, 3rd aerobic tank) | 5 | 5 | 4 | 5 |
| InflowRate | 4 | 4 | 5 | 4 |
| DO (2nd aerobic tank) | 5 | 4 | 5 | 4 |
| Ammonium (biotank effluent) | 4 | 3 | 4 | 3 |
| Nitrate (biotank effluent) | 3 | 2 | 4 | 3 |
| Aeration Power2 | 4 | 3 | 4 | 3 |
| Aeration On/Off | 4 | 3 | 4 | 3 |
| S3N2O (mechanistic sim, 2nd aerobic tank) | 4 | 4 | 2 | 1 |
| S2N2O (mechanistic sim, 1st aerobic tank) | 4 | 4 | 2 | 1 |
| S3NO2 / S3NO / S3NH2OH (mechanistic) | 4 | 3 | 2 | 1 |

Mechanistic simulation variables are down-rated on interpretability and redundancy because they act as model-derived proxies rather than measured process drivers, and are likely collinear with each other and with $N_2O$ dynamics. (An2021 lines 20-30)

**Selected 7 features (exactly)**

1) **S3N2O**
2) **InflowRate**
3) **DO**
4) **Ammonium**
5) **Nitrate**
6) **Aeration Power2**
7) **Aeration On/Off**

These choices prioritize the strongest empirically supported predictors for LSTM $N_2O$ forecasting—autoregressive $N_2O$, influent flow, nitrogen species, and aerobic DO/aeration signals—while avoiding mechanistic-simulation surrogates to reduce leakage/collinearity and

preserve process-driven interpretability. (Pan2024 lines 0-18, Pan2024 lines 107-122, Pan2024 lines 122-128)

**Rationale and expected performance impact**

**S3N2O** captures short-term autocorrelation and fast emission dynamics, typically the dominant contributor at short horizons. (Pan2024 lines 0-18, Pan2024 lines 107-122) **InflowRate** represents hydraulic/load forcing that shifts oxygen demand and nitrogen conversions, becoming more important as horizon increases. (Pan2024 lines 122-128) **DO** and **Aeration Power2 / Aeration On/Off** encode aeration regime and oxygen availability in the emitting tank, consistent with nitrification-linked and AOB-mediated $N_2O$ pathways and with prior feature-importance results emphasizing DO and operational drivers. (Pan2024 lines 0-18, Pan2024 lines 107-122) **Ammonium** and **Nitrate** provide state information on nitrification/denitrification progress and substrate availability, complementing DO/aeration and improving identifiability of emission regimes beyond pure autoregression. (Pan2024 lines 122-128)

**Note:** Performance degradation at longer horizons can indicate missing biochemical/microbial drivers; if available in the table, such variables would be candidates, but feature selection here is constrained to the provided list. (Pan2024 lines 107-122, Pan2024 lines 122-128)

References


1. (Pan2024 lines 0-18): Pan, Y., Seshan, H., Massara, T. M., Daelman, M. R. J., Gruber, W., Kosonen, H., Guo, L., Vanrolleghem, P. A., & Mampaey, K. "Forecasting Nitrous Oxide Emissions from a Full-Scale Wastewater Treatment Plant Using LSTM-Based Deep Learning Models." *Water Research*, vol. 2024, 2024, p. 122754. doi:10.1016/j.watres.2024.122754.

2. (Pan2024 lines 107-122): Pan, Y., Seshan, H., Massara, T. M., Daelman, M. R. J., Gruber, W., Kosonen, H., Guo, L., Vanrolleghem, P. A., & Mampaey, K. "Forecasting Nitrous Oxide Emissions from a Full-Scale Wastewater Treatment Plant Using LSTM-Based Deep Learning Models." *Water Research*, vol. 2024, 2024, p. 122754. doi:10.1016/j.watres.2024.122754.

3. (Pan2024 lines 55-72): Pan, Y., Seshan, H., Massara, T. M., Daelman, M. R. J., Gruber, W., Kosonen, H., Guo, L., Vanrolleghem, P. A., & Mampaey, K. "Forecasting Nitrous Oxide Emissions from a Full-Scale Wastewater Treatment Plant Using LSTM-Based Deep Learning Models." *Water Research*, vol. 2024, 2024, p. 122754. doi:10.1016/j.watres.2024.122754.

4. (An2021 lines 20-30): "An Integrated First Principal and Deep Learning Approach for Modeling Nitrous Oxide Emissions from Wastewater Treatment Plants." *Environmental Science & Technology*, vol. 55, no. 21, 2021, pp. 14450-14460. DOI: 10.1021/acs.est.1c05020.

5. (Pan2024 lines 122-128): Pan, Y., Seshan, H., Massara, T. M., Daelman, M. R. J., Gruber, W., Kosonen, H., Guo, L., Vanrolleghem, P. A., & Mampaey, K. "Forecasting

Nitrous Oxide Emissions from a Full-Scale Wastewater Treatment Plant Using LSTM-Based Deep Learning Models." *Water Research*, vol. 2024, 2024, p. 122754. doi:10.1016/j.watres.2024.122754.

**Claude-Report 1**

**Feature Rating Matrix**

| Feature | $N_2O$ Pathway Relevance | Temporal Predictive Value | Process Interpretability | Redundancy (lower=better) | Correlation with $N_2O$ |
|---|---|---|---|---|---|
| S2N2O | 5 | 5 | 5 | 3 | 0.706 |
| S3N2O | 5 | 5 | 5 | 3 | 0.677 |
| S3NH | 5 | 4 | 5 | 3 | 0.614 |
| S3NO | 4 | 4 | 4 | 3 | 0.668 |
| InflowRate | 4 | 5 | 5 | 1 | 0.394 |
| DO | 4 | 5 | 5 | 1 | −0.415 |
| Aeration On/Off | 3 | 4 | 4 | 2 | 0.274 |
| S3NO3 | 4 | 4 | 4 | 3 | −0.542 |
| S2NH | 4 | 4 | 4 | 4 | 0.617 |
| S1NH | 4 | 3 | 4 | 3 | 0.688 |

**Selected 7 Features**

**S3N2O, S3NH, S3NO, DO, InflowRate, Aeration On/Off, S3NO3**

**Justification**

The target is liquid-phase $N_2O$ in the second aerobic tank (Tank 3). Literature consistently identifies past $N_2O$ emissions, influent flowrate, $NH_4^+$, $NO_x$, and dissolved oxygen as the most significant inputs for LSTM-based $N_2O$ forecasting models (Pan2024 lines 107-122, Pan2024 lines 0-18). These variables are linked to the fast nitrification process and AOB-driven $N_2O$ production pathways (Pan2024 lines 107-122).

**S3N2O** (mechanistic simulation of $N_2O$ in the second aerobic tank) serves as the proxy for past $N_2O$ emissions—the single most important feature identified in the literature—and exhibits the highest same-tank correlation (r = 0.677) with the target (Pan2024 lines 0-18). **S3NH** represents ammonium in the target tank, directly governing AOB-mediated nitrifier denitrification and incomplete hydroxylamine oxidation, both primary $N_2O$ production pathways (Pan2024 lines 107-122). **S3NO** captures nitric oxide, a direct precursor to $N_2O$ in the nitrifier denitrification pathway, with strong correlation (r = 0.668). **DO** in the second

aerobic tank is a critical regulator of $N_2O$ production; low DO promotes incomplete nitrification and elevated $N_2O$ emissions, and its strong negative correlation ($r = -0.415$) confirms its importance (Pan2024 lines 107-122, Pan2024 lines 0-18). **InflowRate** reflects hydraulic loading dynamics that drive transient substrate availability and has been consistently identified as a key input (Pan2024 lines 107-122, Pan2024 lines 0-18, Kim2020 lines 0-16). **Aeration On/Off** captures the intermittent aeration regime in the second aerobic tank, which creates the dynamic oxic/anoxic transitions known to trigger $N_2O$ production spikes; this feature provides temporal pattern information valuable for LSTM sequence learning. **S3NO3** represents nitrate in the target tank ($r = -0.542$), completing the $NO_x$ representation identified as a key input in the literature and capturing the endpoint of nitrification, which inversely relates to incomplete oxidation pathways producing $N_2O$ (Pan2024 lines 107-122, Pan2024 lines 0-18).

This selection prioritizes Tank 3 (second aerobic tank) mechanistic variables to maximize spatial relevance, supplements them with directly measured operational variables (DO, InflowRate, Aeration On/Off), and minimizes redundancy by avoiding highly correlated Tank 2 surrogates (e.g., S2NH, S2NO) when Tank 3 equivalents are available (An2021 lines 30-42, An2021 lines 20-30).

References


1. (Pan2024 lines 107-122): Pan, Y., Seshan, H., Massara, T. M., Daelman, M. R. J., Gruber, W., Kosonen, H., Guo, L., Vanrolleghem, P. A., & Mampaey, K. "Forecasting Nitrous Oxide Emissions from a Full-Scale Wastewater Treatment Plant Using LSTM-Based Deep Learning Models." *Water Research*, vol. 2024, 2024, p. 122754. doi:10.1016/j.watres.2024.122754.

2. (Pan2024 lines 0-18): Pan, Y., Seshan, H., Massara, T. M., Daelman, M. R. J., Gruber, W., Kosonen, H., Guo, L., Vanrolleghem, P. A., & Mampaey, K. "Forecasting Nitrous Oxide Emissions from a Full-Scale Wastewater Treatment Plant Using LSTM-Based Deep Learning Models." *Water Research*, vol. 2024, 2024, p. 122754. doi:10.1016/j.watres.2024.122754.

3. (Kim2020 lines 0-16): Kim, J., et al. "Integrated Model for Understanding N2O Emissions from Wastewater Treatment Plants: A Deep Learning Approach." *Environmental Science & Technology*, vol. 54, no. 24, 2020, pp. 15716-15725. DOI: 10.1021/acs.est.0c05231.

4. (An2021 lines 30-42): "An Integrated First Principal and Deep Learning Approach for Modeling Nitrous Oxide Emissions from Wastewater Treatment Plants." *Environmental Science & Technology*, vol. 55, no. 21, 2021, pp. 14450-14460. DOI: 10.1021/acs.est.1c05020.

5. (An2021 lines 20-30): "An Integrated First Principal and Deep Learning Approach for Modeling Nitrous Oxide Emissions from Wastewater Treatment Plants." *Environmental Science & Technology*, vol. 55, no. 21, 2021, pp. 14450-14460. DOI: 10.1021/acs.est.1c05020.

**Claude-Report 2**

**Feature Selection Report for LSTM-Based $N_2O$ Emission Modeling**

**Feature Rating Matrix**

| Feature | $N_2O$ Pathway Relevance | Temporal Predictive Value | Process Interpretability | Redundancy (lower=better) | Correlation with Target | Overall Score |
|---|---|---|---|---|---|---|
| S2N2O | 5 | 5 | 5 | 3 | 0.706 | 4.5 |
| S3N2O | 5 | 5 | 5 | 3 | 0.677 | 4.5 |
| S1NH | 4 | 4 | 4 | 3 | 0.688 | 3.8 |
| InflowRate | 5 | 5 | 5 | 5 | 0.394 | 5.0 |
| DO | 5 | 5 | 5 | 4 | -0.415 | 4.8 |
| S2NO3 | 4 | 4 | 4 | 3 | -0.544 | 3.8 |
| S3NO | 4 | 4 | 4 | 3 | 0.668 | 3.8 |
| Aeration On/Off | 4 | 4 | 5 | 4 | 0.274 | 4.3 |
| S2NH | 4 | 4 | 4 | 2 | 0.617 | 3.5 |
| S3NH | 4 | 4 | 4 | 2 | 0.614 | 3.5 |
| Aeration Power2 | 4 | 4 | 5 | 3 | 0.265 | 4.0 |
| S2NO | 4 | 4 | 4 | 2 | 0.677 | 3.5 |
| S3O | 4 | 4 | 4 | 2 | -0.375 | 3.5 |

**Selected 7 Features**

**1. InflowRate** — Influent flowrate is a primary driver of $N_2O$ dynamics, governing hydraulic loading and substrate availability. It was identified as a key input in both deep learning and integrated ASM-LSTM modeling frameworks (Kim2020 lines 0-16, An2021 lines 30-42, Pan2024 lines 107-122). Its correlation with $N_2O$ (r = 0.394) and low redundancy with mechanistic variables make it indispensable.

**2. DO** — Dissolved oxygen in the 2nd aerobic tank directly governs AOB-mediated $N_2O$ production via the nitrifier denitrification and hydroxylamine oxidation pathways. DO was consistently identified as a critical feature across multiple studies (Kim2020 lines 0-16, Kim2020 lines 16-30, Pan2024 lines 107-122). Its strong negative correlation (r = -0.415) reflects the well-established inverse relationship between DO and $N_2O$ emissions.

**3. S2N2O** — The mechanistic simulation of $N_2O$ in the 1st aerobic tank (upstream of the target tank) provides the strongest correlation with the target (r = 0.706). Integrated ASM-LSTM approaches demonstrated that mechanistic model outputs substantially improve deep learning predictions (An2021 lines 30-42).

**4. S3N2O** — The mechanistic $N_2O$ estimate for the 2nd aerobic tank (the target tank itself) captures the process-model baseline (r = 0.677), providing the LSTM with a physics-informed prior that it can correct for residual dynamics (An2021 lines 30-42).

**5. S3NO** — Nitric oxide in the target tank is a direct intermediate in the nitrifier denitrification $N_2O$ production pathway (r = 0.668). $NH_4^+$ and NOx species were identified as significant inputs for $N_2O$ forecasting models (Pan2024 lines 107-122, Pan2024 lines 0-18).

**6. Aeration On/Off** — The intermittent aeration regime in the 2nd aerobic tank creates transient DO conditions that are primary triggers for $N_2O$ emission peaks. This feature captures the temporal cycling pattern critical for LSTM sequence learning (r = 0.274), complementing the instantaneous DO measurement with operational state information (Kim2020 lines 0-16, Kim2020 lines 16-30).

**7. S1NH** — Ammonium concentration from the anaerobic tank mechanistic simulation (r = 0.688) represents the substrate loading entering the aerobic zone. Ammonium was consistently ranked among the most influential features for $N_2O$ prediction (Pan2024 lines 107-122, Pan2024 lines 0-18), and using the upstream mechanistic estimate avoids redundancy with the effluent ammonium sensor while capturing influent nitrogen dynamics (An2021 lines 30-42).

**Additional Notes**

The selected features span three categories: measured operational variables (InflowRate, DO, Aeration On/Off), upstream mechanistic outputs (S1NH, S2N2O), and target-tank mechanistic outputs (S3N2O, S3NO). This hybrid approach mirrors the integrated ASM-LSTM framework that demonstrated superior performance over purely data-driven models (An2021 lines 30-42). Features such as S2NH and S3NH were excluded due to high redundancy with S1NH and the mechanistic $N_2O$ variables. The inclusion of both S2N2O and S3N2O provides the LSTM with spatial gradient information along the plug-flow reactor, which is expected to improve temporal forecasting accuracy.

References


1. (Kim2020 lines 0-16): Kim, J., et al. "Integrated Model for Understanding N2O Emissions from Wastewater Treatment Plants: A Deep Learning Approach." *Environmental Science & Technology*, vol. 54, no. 24, 2020, pp. 15716-15725. DOI: 10.1021/acs.est.0c05231.

2. (An2021 lines 30-42): "An Integrated First Principal and Deep Learning Approach for Modeling Nitrous Oxide Emissions from Wastewater Treatment Plants." *Environmental Science & Technology*, vol. 55, no. 21, 2021, pp. 14450-14460. DOI: 10.1021/acs.est.1c05020.

3. (Pan2024 lines 107-122): Pan, Y., Seshan, H., Massara, T. M., Daelman, M. R. J., Gruber, W., Kosonen, H., Guo, L., Vanrolleghem, P. A., & Mampaey, K. "Forecasting Nitrous Oxide Emissions from a Full-Scale Wastewater Treatment Plant Using LSTM-Based Deep Learning Models." *Water Research*, vol. 2024, 2024, p. 122754. doi:10.1016/j.watres.2024.122754.

4. (Kim2020 lines 16-30): Kim, J., et al. "Integrated Model for Understanding N2O Emissions from Wastewater Treatment Plants: A Deep Learning Approach." *Environmental Science & Technology*, vol. 54, no. 24, 2020, pp. 15716-15725. DOI: 10.1021/acs.est.0c05231.

5. (Pan2024 lines 0-18): Pan, Y., Seshan, H., Massara, T. M., Daelman, M. R. J., Gruber, W., Kosonen, H., Guo, L., Vanrolleghem, P. A., & Mampaey, K. "Forecasting Nitrous Oxide Emissions from a Full-Scale Wastewater Treatment Plant Using LSTM-Based Deep Learning Models." *Water Research*, vol. 2024, 2024, p. 122754. doi:10.1016/j.watres.2024.122754.

**Claude-Report 3**

**Correlation with target (N2O)**

| Feature | Corr | Feature | Corr | Feature | Corr |
|---|---|---|---|---|---|
| N2O | 1.000000 | S2NH2OH | 0.474384 | X1H | 0.138615 |
| S2N2O | 0.705884 | S3NH2OH | 0.460670 | S1NO2 | 0.077573 |
| S1NH | 0.687888 | S2NO2 | 0.449812 | X2H | 0.072881 |
| S3N2O | 0.676676 | S3NO2 | 0.447637 | X3H | 0.072420 |
| S2NO | 0.676656 | S2S | 0.426502 | X2I | 0.059011 |
| S3NO | 0.668339 | S3S | 0.411483 | X3I | 0.053487 |
| S2NH | 0.616876 | InflowRate | 0.394305 | Ammonium | 0.029851 |
| S3NH | 0.614368 | RAS_F | 0.384918 | X2AOB | 0.023124 |
| S1NH2OH | 0.600702 | RAS_CDE | 0.347648 | X3AOB | 0.021018 |

**Feature Rating Matrix**

| Feature | Relevance to $N_2O$ Pathways | Temporal Predictive Value (LSTM) | Process Interpretability | Redundancy (lower = better) | Correlation with Target |
|---|---|---|---|---|---|
| S2N2O | 5 | 5 | 5 | 3 (correlated with S3N2O) | 0.706 |
| S3N2O | 5 | 5 | 5 | 3 (correlated with S2N2O) | 0.677 |
| S1NH | 4 | 4 | 4 | 2 | 0.688 |

| Feature | Relevance to $N_2O$ Pathways | Temporal Predictive Value (LSTM) | Process Interpretability | Redundancy (lower = better) | Correlation with Target |
|---|---|---|---|---|---|
| S2NO | 4 | 4 | 4 | 3 (correlated with S3NO) | 0.677 |
| S3NO | 4 | 4 | 4 | 3 (correlated with S2NO) | 0.668 |
| S2NH | 4 | 4 | 4 | 3 (correlated with S3NH) | 0.617 |
| S3NH | 4 | 4 | 4 | 3 (correlated with S2NH) | 0.614 |
| InflowRate | 4 | 5 | 5 | 1 | 0.394 |
| DO | 4 | 5 | 5 | 1 | −0.415 |
| Aeration Power2 | 3 | 4 | 4 | 2 (related to DO) | 0.265 |
| Aeration On/Off | 3 | 4 | 4 | 2 (related to DO) | 0.274 |
| S2NO3 | 3 | 3 | 4 | 2 | −0.544 |
| S2O | 3 | 3 | 4 | 3 (related to DO) | −0.503 |
| S1NH2OH | 3 | 3 | 3 | 2 | 0.601 |
| S1N2 | 2 | 3 | 3 | 1 | −0.685 |

**Selected 7 Features**

**S2N2O, S3NH, InflowRate, DO, Aeration Power2, S1NH, S2NO3**

**Justification**

The selected features were chosen to maximize predictive performance while minimizing redundancy and maintaining process interpretability for liquid-phase $N_2O$ modeling in the second aerobic tank.

1. **S2N2O** (mechanistic $N_2O$ simulation, 1st aerobic tank): This feature has the highest correlation with the target (r = 0.706) and directly represents the upstream $N_2O$ concentration entering the second aerobic tank, providing critical temporal context for the LSTM to learn $N_2O$ transport and production dynamics.

2. **S3NH** (mechanistic $NH_4^+$ simulation, 2nd aerobic tank): Ammonium in the target tank is a direct substrate for ammonia-oxidizing bacteria (AOB), which are the primary producers of $N_2O$ via the hydroxylamine oxidation and nitrifier denitrification pathways (Pan2024 lines 107-122). Its correlation with the target is 0.614.

3. **InflowRate**: Influent flow rate was identified as a key driver of $N_2O$ emissions in both Kim et al. and Pan et al., reflecting hydraulic loading dynamics that influence substrate availability and retention time (Kim2020 lines 0-16, Pan2024 lines 107-122). Its temporal variability provides strong sequential information for LSTM learning.

4. **DO** (dissolved oxygen, 2nd aerobic tank): DO is a well-established control variable for $N_2O$ production, as low DO conditions promote nitrifier denitrification by AOB (Kim2020 lines 16-30, Pan2024 lines 0-18). Its strong negative correlation ($r = -0.415$) confirms its inverse relationship with $N_2O$ emissions.

5. **Aeration Power2** (aeration power, 2nd aerobic tank): This variable captures the aeration control dynamics directly in the target tank and complements DO by providing information about the physical stripping rate of $N_2O$ from the liquid phase. Aeration valve positions and air flow rates were used as key inputs in prior LSTM-based $N_2O$ models (Pan2024 lines 72-85, Kim2020 lines 0-16).

6. **S1NH** (mechanistic $NH_4^+$ simulation, anaerobic tank): With the second-highest correlation ($r = 0.688$), this feature captures upstream ammonium loading from the anaerobic zone, providing the LSTM with leading temporal information about substrate availability that will propagate through the aerobic tanks.

7. **S2NO3** (mechanistic $NO_3^-$ simulation, 1st aerobic tank): Nitrate in the upstream aerobic tank has a strong negative correlation ($r = -0.544$) with $N_2O$, reflecting the competition between complete nitrification and incomplete pathways that generate $N_2O$. Including NOx-related variables was identified as important for capturing the nitrification process dynamics (Pan2024 lines 107-122).

**Additional Notes**

The selection deliberately avoids including both S2N2O and S3N2O simultaneously to reduce redundancy (r between them is expected to be very high). Similarly, S2NH and S3NH are highly correlated, so only S3NH (local to the target tank) and S1NH (upstream, providing lead-time information) were retained. The combination of mechanistic model outputs with measured operational variables (InflowRate, DO, Aeration Power2) provides the LSTM with both process-state information and control-action signals, which is expected to improve forecasting across multiple prediction horizons (Pan2024 lines 72-85, Pan2024 lines 107-122).

References

8. (Pan2024 lines 107-122): Pan, Y., Seshan, H., Massara, T. M., Daelman, M. R. J., Gruber, W., Kosonen, H., Guo, L., Vanrolleghem, P. A., & Mampaey, K. "Forecasting Nitrous Oxide Emissions from a Full-Scale Wastewater Treatment Plant Using LSTM-

Based Deep Learning Models." *Water Research*, vol. 2024, 2024, p. 122754. doi:10.1016/j.watres.2024.122754.

9. (Kim2020 lines 0-16): Kim, J., et al. "Integrated Model for Understanding N2O Emissions from Wastewater Treatment Plants: A Deep Learning Approach." *Environmental Science & Technology*, vol. 54, no. 24, 2020, pp. 15716-15725. DOI: 10.1021/acs.est.0c05231.

10. (Kim2020 lines 16-30): Kim, J., et al. "Integrated Model for Understanding N2O Emissions from Wastewater Treatment Plants: A Deep Learning Approach." *Environmental Science & Technology*, vol. 54, no. 24, 2020, pp. 15716-15725. DOI: 10.1021/acs.est.0c05231.

11. (Pan2024 lines 0-18): Pan, Y., Seshan, H., Massara, T. M., Daelman, M. R. J., Gruber, W., Kosonen, H., Guo, L., Vanrolleghem, P. A., & Mampaey, K. "Forecasting Nitrous Oxide Emissions from a Full-Scale Wastewater Treatment Plant Using LSTM-Based Deep Learning Models." *Water Research*, vol. 2024, 2024, p. 122754. doi:10.1016/j.watres.2024.122754.

12. (Pan2024 lines 72-85): Pan, Y., Seshan, H., Massara, T. M., Daelman, M. R. J., Gruber, W., Kosonen, H., Guo, L., Vanrolleghem, P. A., & Mampaey, K. "Forecasting Nitrous Oxide Emissions from a Full-Scale Wastewater Treatment Plant Using LSTM-Based Deep Learning Models." *Water Research*, vol. 2024, 2024, p. 122754. doi:10.1016/j.watres.2024.122754.

**Claude-Report 4**

**Feature Rating Matrix**

| Feature | Relevance to $N_2O$ Pathways | Temporal Predictive Value (LSTM) | Process Interpretability | Redundancy (lower = better) | Correlation with Target |
|---|---|---|---|---|---|
| S2N2O | 5 | 5 | 5 | 3 (correlated with S3N2O) | 0.706 |
| S3N2O | 5 | 5 | 5 | 3 (correlated with S2N2O) | 0.677 |
| S1NH | 4 | 4 | 4 | 2 | 0.688 |
| S2NO | 4 | 4 | 4 | 3 | 0.677 |
| S3NO | 4 | 4 | 4 | 3 (correlated with S2NO) | 0.668 |
| S2NH | 4 | 4 | 4 | 3 (correlated with S1NH) | 0.617 |

| Feature | Relevance to $N_2O$ Pathways | Temporal Predictive Value (LSTM) | Process Interpretability | Redundancy (lower = better) | Correlation with Target |
|---|---|---|---|---|---|
| S3NH | 4 | 4 | 4 | 4 (correlated with S2NH, S1NH) | 0.614 |
| InflowRate | 5 | 5 | 5 | 1 | 0.394 |
| DO | 5 | 5 | 5 | 1 | −0.415 |
| Aeration Power2 | 4 | 4 | 4 | 3 (related to DO) | 0.265 |
| Aeration On/Off | 4 | 4 | 4 | 3 (related to DO) | 0.274 |
| S2NO3 | 4 | 3 | 4 | 2 | −0.544 |
| S2O | 3 | 3 | 4 | 3 (related to DO) | −0.503 |
| S1NH2OH | 3 | 3 | 3 | 2 | 0.601 |

**Selected 7 Features**

**S2N2O, InflowRate, DO, S1NH, S2NO3, Aeration On/Off, S3NH**

**Justification**

1. **S2N2O** (r = 0.706): This mechanistic simulation of $N_2O$ in the first aerobic tank is the strongest single predictor of the target. It captures upstream $N_2O$ dynamics that directly propagate to the second aerobic tank, providing the LSTM with critical temporal context analogous to the role of past $N_2O$ emissions identified as the most significant input in LSTM-based forecasting models (Pan2024 lines 107-122, Pan2024 lines 0-18).
2. **InflowRate** (r = 0.394): Influent flow rate governs hydraulic loading and substrate delivery, identified as a key driver of $N_2O$ emissions in both Kim et al. and Pan et al. studies (Kim2020 lines 0-16, Kim2020 lines 16-30, Pan2024 lines 107-122). Its dynamic fluctuations are well-suited for LSTM temporal learning.
3. **DO** (r = −0.415): Dissolved oxygen in the second aerobic tank directly modulates AOB-driven $N_2O$ production via the nitrifier denitrification pathway. DO was consistently identified as a critical input across multiple LSTM-based $N_2O$ models (Kim2020 lines 16-30, Pan2024 lines 107-122, Pan2024 lines 72-85).
4. **S1NH** (r = 0.688): Ammonium concentration in the anaerobic tank represents the substrate supply for downstream nitrification, the primary $N_2O$-generating process. Ammonium was identified as a key feature in prior studies (Kim2020 lines 0-16,

Pan2024 lines 107-122). Selecting S1NH over S2NH or S3NH provides upstream information with lower redundancy relative to the target tank.

5. **S2NO3** (r = −0.544): Nitrate in the first aerobic tank reflects nitrification completion and denitrification potential. Its strong negative correlation with $N_2O$ indicates that higher nitrate accumulation corresponds to conditions suppressing $N_2O$, consistent with the role of NOx as a key input (Pan2024 lines 107-122). This complements S2NO by capturing a different nitrogen species without excessive redundancy.

6. **Aeration On/Off** (r = 0.274): The intermittent aeration pattern of the second aerobic tank creates transient DO conditions that are well-established drivers of $N_2O$ production during aerobic-anoxic transitions. This feature is analogous to the aeration valve positions used in Pan et al. (Pan2024 lines 72-85) and provides temporal pattern information that LSTMs can exploit effectively.

7. **S3NH** (r = 0.614): Ammonium in the target tank (second aerobic) directly reflects the local substrate availability for nitrification-coupled $N_2O$ production. Including this alongside S1NH captures both upstream loading and local conditions, consistent with the importance of $NH_4^+$ in AOB-related $N_2O$ pathways (Pan2024 lines 107-122).

**Additional Notes**

The selected features balance measured operational variables (InflowRate, DO, Aeration On/Off) with mechanistic model outputs (S2N2O, S1NH, S2NO3, S3NH), providing the LSTM with both direct process measurements and physically informed state estimates. S2N2O serves as a proxy for historical $N_2O$ dynamics, which was identified as the most influential input for LSTM-based $N_2O$ forecasting (Pan2024 lines 0-18). Redundancy was minimized by avoiding simultaneous selection of highly correlated pairs (e.g., S2NH/S3NH/S1NH—only S1NH and S3NH retained; S2NO/S3NO—excluded in favor of S2NO3). This feature set is expected to capture the dominant $N_2O$ production mechanisms: nitrifier denitrification (DO, aeration dynamics, ammonium) and incomplete nitrification/denitrification (nitrate, flow dynamics).

References

1. (Pan2024 lines 107-122): Pan, Y., Seshan, H., Massara, T. M., Daelman, M. R. J., Gruber, W., Kosonen, H., Guo, L., Vanrolleghem, P. A., & Mampaey, K. "Forecasting Nitrous Oxide Emissions from a Full-Scale Wastewater Treatment Plant Using LSTM-Based Deep Learning Models." *Water Research*, vol. 2024, 2024, p. 122754. doi:10.1016/j.watres.2024.122754.

2. (Pan2024 lines 0-18): Pan, Y., Seshan, H., Massara, T. M., Daelman, M. R. J., Gruber, W., Kosonen, H., Guo, L., Vanrolleghem, P. A., & Mampaey, K. "Forecasting Nitrous Oxide Emissions from a Full-Scale Wastewater Treatment Plant Using LSTM-Based Deep Learning Models." *Water Research*, vol. 2024, 2024, p. 122754. doi:10.1016/j.watres.2024.122754.

3. (Kim2020 lines 0-16): Kim, J., et al. "Integrated Model for Understanding N2O Emissions from Wastewater Treatment Plants: A Deep Learning Approach." *Environmental Science & Technology*, vol. 54, no. 24, 2020, pp. 15716-15725. DOI: 10.1021/acs.est.0c05231.
4. (Kim2020 lines 16-30): Kim, J., et al. "Integrated Model for Understanding N2O Emissions from Wastewater Treatment Plants: A Deep Learning Approach." *Environmental Science & Technology*, vol. 54, no. 24, 2020, pp. 15716-15725. DOI: 10.1021/acs.est.0c05231.
5. (Pan2024 lines 72-85): Pan, Y., Seshan, H., Massara, T. M., Daelman, M. R. J., Gruber, W., Kosonen, H., Guo, L., Vanrolleghem, P. A., & Mampaey, K. "Forecasting Nitrous Oxide Emissions from a Full-Scale Wastewater Treatment Plant Using LSTM-Based Deep Learning Models." *Water Research*, vol. 2024, 2024, p. 122754. doi:10.1016/j.watres.2024.122754.

**Claude-Report 5**

**Feature Rating Matrix**

| Feature | Relevance to $N_2O$ Pathways | Temporal Predictive Value | Process Interpretability | Redundancy (lower = better) | Correlation with Target |
|---|---|---|---|---|---|
| S2N2O | 5 | 5 | 5 | 3 (correlated with S3N2O) | 0.706 |
| S3N2O | 5 | 5 | 5 | 3 (correlated with S2N2O) | 0.677 |
| S1NH | 4 | 4 | 4 | 2 | 0.688 |
| S2NO | 4 | 4 | 4 | 3 (correlated with S3NO) | 0.677 |
| S3NO | 4 | 4 | 4 | 3 (correlated with S2NO) | 0.668 |
| S2NH | 4 | 4 | 4 | 3 (correlated with S3NH) | 0.617 |
| S3NH | 4 | 4 | 4 | 3 (correlated with S2NH) | 0.614 |
| InflowRate | 5 | 5 | 5 | 1 | 0.394 |
| DO | 5 | 5 | 5 | 1 | −0.415 |
| Aeration Power2 | 4 | 4 | 4 | 3 (related to DO) | 0.265 |

| Feature | Relevance to $N_2O$ Pathways | Temporal Predictive Value | Process Interpretability | Redundancy (lower = better) | Correlation with Target |
|---|---|---|---|---|---|
| Aeration On/Off | 4 | 4 | 4 | 3 (related to DO) | 0.274 |
| S2NO3 | 4 | 3 | 4 | 2 | −0.544 |
| S3NO3 | 4 | 3 | 4 | 3 (correlated with S2NO3) | −0.542 |
| S2O | 3 | 3 | 4 | 3 (related to DO) | −0.503 |
| S1N2 | 3 | 3 | 3 | 1 | −0.685 |

**Selected 7 Features**

1. **S3N2O** — Mechanistic simulation of $N_2O$ in the 2nd aerobic tank (the target tank itself). Past $N_2O$ emissions have been identified as the most significant input for LSTM-based $N_2O$ forecasting models, providing critical autoregressive information that captures temporal dynamics (Pan2024 lines 0-18, Pan2024 lines 107-122). This variable serves as a mechanistic proxy for historical $N_2O$ state in the target compartment (correlation: 0.677).

2. **InflowRate** — Influent flow rate to the biotank. Influent flowrate was consistently identified as a key driver of $N_2O$ emissions in both Kim et al. and Pan et al., ranking among the most important features in sensitivity and feature importance analyses (Kim2020 lines 0-16, Kim2020 lines 16-30, Pan2024 lines 107-122). It governs hydraulic loading and substrate availability, directly influencing nitrification dynamics.

3. **DO** — Dissolved oxygen in the 2nd aerobic tank. DO is a primary control variable governing AOB-mediated $N_2O$ production pathways; low DO conditions promote incomplete nitrification and elevated $N_2O$ emissions (Kim2020 lines 16-30, Pan2024 lines 107-122). Its strong negative correlation (−0.415) with the target confirms its regulatory role.

4. **S2NH** — Mechanistic simulation of ammonium in the 1st aerobic tank. Ammonium is a direct substrate for nitrification and AOB-driven $N_2O$ production. $NH_4^+$ was identified as a key feature in multiple LSTM-based $N_2O$ models (Kim2020 lines 0-16, Pan2024 lines 107-122). S2NH captures upstream ammonium dynamics feeding into the target tank (correlation: 0.617).

5. **S2N2O** — Mechanistic simulation of $N_2O$ in the 1st aerobic tank. This provides upstream $N_2O$ concentration information, which is highly correlated with the target (0.706) and captures the propagation of $N_2O$ production from the preceding aerobic compartment, analogous to how internal recycles transfer species between compartments (Pan2024 lines 72-85).

6. **S3NO3** — Mechanistic simulation of nitrate in the 2nd aerobic tank. Nitrate reflects the extent of complete nitrification and denitrification activity. NOx species (nitrate/nitrite) were identified as significant model inputs for $N_2O$ prediction (Pan2024 lines 107-122, Kim2020 lines 0-16). The strong negative correlation (−0.542) indicates an inverse relationship with $N_2O$, consistent with conditions where incomplete nitrification favors $N_2O$ over $NO_3^-$ production.

7. **Aeration Power2** — Aeration power of the 2nd aerobic tank. Air flow rate was used as a core input feature in the Kim et al. study for $N_2O$ modeling (Kim2020 lines 0-16, Kim2020 lines 16-30), and aeration valve positions were similarly used by Pan et al. (Pan2024 lines 72-85). Aeration Power2 directly controls oxygen transfer in the target tank and provides complementary information to DO, capturing the dynamic aeration control signal rather than the resulting oxygen concentration.

**Summary**

The selected features span the key $N_2O$ production drivers: substrate availability (S2NH), oxygen regime (DO, Aeration Power2), nitrification products (S3NO3), upstream and local $N_2O$ state (S2N2O, S3N2O), and hydraulic loading (InflowRate). This selection minimizes redundancy while covering the AOB-mediated $N_2O$ pathway variables identified as most influential in prior LSTM-based modeling studies (Pan2024 lines 107-122, Kim2020 lines 16-30). The inclusion of mechanistic simulation variables from both the target and upstream tanks provides the LSTM with spatiotemporal context across compartments, which is expected to improve sequence-to-sequence prediction performance.

References

1. (Pan2024 lines 0-18): Pan, Y., Seshan, H., Massara, T. M., Daelman, M. R. J., Gruber, W., Kosonen, H., Guo, L., Vanrolleghem, P. A., & Mampaey, K. “Forecasting Nitrous Oxide Emissions from a Full-Scale Wastewater Treatment Plant Using LSTM-Based Deep Learning Models.” *Water Research*, vol. 2024, 2024, p. 122754. doi:10.1016/j.watres.2024.122754.

2. (Pan2024 lines 107-122): Pan, Y., Seshan, H., Massara, T. M., Daelman, M. R. J., Gruber, W., Kosonen, H., Guo, L., Vanrolleghem, P. A., & Mampaey, K. “Forecasting Nitrous Oxide Emissions from a Full-Scale Wastewater Treatment Plant Using LSTM-Based Deep Learning Models.” *Water Research*, vol. 2024, 2024, p. 122754. doi:10.1016/j.watres.2024.122754.

3. (Kim2020 lines 0-16): Kim, J., et al. “Integrated Model for Understanding N2O Emissions from Wastewater Treatment Plants: A Deep Learning Approach.” *Environmental Science & Technology*, vol. 54, no. 24, 2020, pp. 15716-15725. DOI: 10.1021/acs.est.0c05231.

4. (Kim2020 lines 16-30): Kim, J., et al. “Integrated Model for Understanding N2O Emissions from Wastewater Treatment Plants: A Deep Learning Approach.”

*Environmental Science & Technology*, vol. 54, no. 24, 2020, pp. 15716-15725. DOI: 10.1021/acs.est.0c05231.

5. (Pan2024 lines 72-85): Pan, Y., Seshan, H., Massara, T. M., Daelman, M. R. J., Gruber, W., Kosonen, H., Guo, L., Vanrolleghem, P. A., & Mampaey, K. "Forecasting Nitrous Oxide Emissions from a Full-Scale Wastewater Treatment Plant Using LSTM-Based Deep Learning Models." *Water Research*, vol. 2024, 2024, p. 122754. doi:10.1016/j.watres.2024.122754.

**Gemini-Report 1**

To simulate liquid-phase $N_2O$ emissions in the second aerobic tank using an LSTM-based model, the following seven features are selected based on their relevance to $N_2O$ production pathways, temporal predictive value, process interpretability, and minimal redundancy.

**Feature Rating Matrix**

| Feature | Relevance to $N_2O$ Production Pathways (1-5) | Temporal Predictive Value for LSTM (1-5) | Process Interpretability (1-5) | Redundancy with other variables (1-5, 5=low redundancy) | Total Score |
|---|---|---|---|---|---|
| S3N2O | 5 | 5 | 5 | 4 | 19 |
| DO | 5 | 4 | 5 | 4 | 18 |
| InflowRate | 4 | 4 | 5 | 5 | 18 |
| S3NH | 5 | 4 | 4 | 4 | 17 |
| S3NO2 | 5 | 4 | 4 | 4 | 17 |
| Aeration Power2 | 4 | 4 | 5 | 4 | 17 |
| S3NO3 | 4 | 4 | 4 | 4 | 16 |

**Selected Features and Rationale**

The best 7 features for the LSTM model are: S3N2O, DO, InflowRate, S3NH, S3NO2, Aeration Power2, and S3NO3.

1. **S3N2O (mechanistic simulation of 2nd aerobic tank N2O):** This feature represents historical liquid-phase $N_2O$ concentrations within the target tank, which is a critical input for LSTM models due to the temporal dependencies of $N_2O$ emissions (An2021 lines 20-30, Pan2024 lines 0-18, Pan2024 lines 107-122). Its high correlation (0.676676) with the target $N_2O$ further supports its inclusion.
2. **DO (DO of 2nd aerobic tank):** Dissolved oxygen is a primary environmental factor influencing $N_2O$ production pathways, particularly nitrification and denitrification

(An2021 lines 20-30, Pan2024 lines 0-18, Pan2024 lines 107-122, An2021 lines 30-42). Direct measurement in the target tank provides immediate process state information.

3. **InflowRate (Influent of the biotank):** Influent flowrate directly impacts hydraulic retention time and substrate loading, which are fundamental drivers of biological processes and $N_2O$ emissions in wastewater treatment (An2021 lines 20-30, Pan2024 lines 0-18, Pan2024 lines 107-122). Its correlation of 0.394305 indicates significant influence.

4. **S3NH (mechanistic simulation of 2nd aerobic tank ammonium):** Ammonium ($NH_4^+$) is the primary substrate for nitrification, a key pathway for $N_2O$ generation (An2021 lines 20-30, Pan2024 lines 0-18, Pan2024 lines 107-122, An2021 lines 30-42). The simulated value specific to the target tank (correlation 0.614368) provides a robust indicator of substrate availability.

5. **S3NO2 (mechanistic simulation of 2nd aerobic tank nitrite):** Nitrite ($NO_2^-$) is a critical intermediate in both nitrification and denitrification, and its accumulation is strongly linked to $N_2O$ production (An2021 lines 20-30, An2021 lines 30-42). The simulated nitrite in the target tank shows a good correlation of 0.447637.

6. **Aeration Power2 (aeration power of the 2nd aerobic tank):** This operational parameter directly controls the DO levels in the target tank, thereby indirectly but significantly influencing $N_2O$ production (Pan2024 lines 0-18, Pan2024 lines 107-122). Its direct control over a critical environmental factor makes it highly interpretable and valuable.

7. **S3NO3 (mechanistic simulation of 2nd aerobic tank nitrate):** Nitrate ($NO_3^-$) is the end product of nitrification and a substrate for denitrification, both of which can produce $N_2O$ (An2021 lines 20-30, An2021 lines 30-42). The simulated nitrate in the target tank exhibits a strong negative correlation (-0.541990), indicating its inverse relationship with $N_2O$ under certain conditions.

These features are expected to improve model performance by providing comprehensive information on the historical $N_2O$ state, key nitrogen species concentrations, critical environmental conditions (DO), and operational controls (InflowRate, Aeration Power2) directly relevant to $N_2O$ formation in the second aerobic tank. The inclusion of simulated profiles (S3N2O, S3NH, S3NO2, S3NO3) leverages mechanistic understanding, which can enhance the deep learning model's ability to capture complex $N_2O$ dynamics (An2021 lines 30-42). The emphasis on process variables over historical $N_2O$ alone is also crucial for robust predictions, especially over longer horizons (Pan2024 lines 0-18).

References

1. (An2021 lines 20-30): "An Integrated First Principal and Deep Learning Approach for Modeling Nitrous Oxide Emissions from Wastewater Treatment Plants."

*Environmental Science & Technology*, vol. 55, no. 21, 2021, pp. 14450-14460. DOI: 10.1021/acs.est.1c05020.

2. (Pan2024 lines 0-18): Pan, Y., Seshan, H., Massara, T. M., Daelman, M. R. J., Gruber, W., Kosonen, H., Guo, L., Vanrolleghem, P. A., & Mampaey, K. "Forecasting Nitrous Oxide Emissions from a Full-Scale Wastewater Treatment Plant Using LSTM-Based Deep Learning Models." *Water Research*, vol. 2024, 2024, p. 122754. doi:10.1016/j.watres.2024.122754.
3. (Pan2024 lines 107-122): Pan, Y., Seshan, H., Massara, T. M., Daelman, M. R. J., Gruber, W., Kosonen, H., Guo, L., Vanrolleghem, P. A., & Mampaey, K. "Forecasting Nitrous Oxide Emissions from a Full-Scale Wastewater Treatment Plant Using LSTM-Based Deep Learning Models." *Water Research*, vol. 2024, 2024, p. 122754. doi:10.1016/j.watres.2024.122754.
4. (An2021 lines 30-42): "An Integrated First Principal and Deep Learning Approach for Modeling Nitrous Oxide Emissions from Wastewater Treatment Plants." *Environmental Science & Technology*, vol. 55, no. 21, 2021, pp. 14450-14460. DOI: 10.1021/acs.est.1c05020.

**Gemini-Report 2**

To simulate liquid-phase $N_2O$ emissions in the second aerobic tank (third tank) using an LSTM-based model, the following seven features are selected based on their relevance to $N_2O$ production pathways, temporal predictive value, process interpretability, and minimal redundancy.

**Feature Rating Matrix**

| Feature | Relevance to $N_2O$ Production Pathways (1-5) | Temporal Predictive Value for LSTM (1-5) | Process Interpretability (1-5) | Redundancy with Other Variables (1-5) |
|---|---|---|---|---|
| S3N2O | 5 | 5 | 5 | 1 |
| DO | 5 | 4 | 5 | 2 |
| S3NH | 5 | 4 | 4 | 1 |
| S3NO2 | 5 | 4 | 4 | 1 |
| S3NO | 5 | 4 | 4 | 2 |
| S3NO3 | 4 | 4 | 4 | 1 |
| InflowRate | 3 | 4 | 5 | 1 |

**Selected 7 Features and Rationale**

1. **S3N2O** (mechanistic simulation of $N_2O$ in 2nd aerobic tank): This feature represents historical liquid-phase $N_2O$ concentrations in the target tank. It is crucial for an LSTM

(Long Short-Term Memory) model due to its strong temporal predictive value and direct relevance to the target variable (An2021 lines 20-30, Pan2024 lines 0-18, Pan2024 lines 107-122, Kim2020 lines 0-16). Its high correlation (0.676676) further supports its inclusion.

2. **DO** (Dissolved Oxygen of 2nd aerobic tank): Dissolved Oxygen is a critical parameter directly influencing nitrification and denitrification pathways, both of which are major sources of $N_2O$ emissions in aerobic tanks (An2021 lines 20-30, An2021 lines 30-42, Pan2024 lines 0-18, Pan2024 lines 107-122, Kim2020 lines 0-16). Its strong negative correlation (-0.415064) highlights its importance.

3. **S3NH** (mechanistic simulation of NH in 2nd aerobic tank): Ammonium ($NH_4^+$) is the primary substrate for nitrification, a key process in $N_2O$ formation (An2021 lines 20-30, An2021 lines 30-42, Pan2024 lines 0-18, Pan2024 lines 107-122, Kim2020 lines 0-16). The simulated value specific to the second aerobic tank (S3NH) is more directly relevant than the general "Ammonium" effluent measurement, with a higher correlation (0.614368).

4. **S3NO2** (mechanistic simulation of $NO_2$ in 2nd aerobic tank): Nitrite ($NO_2^-$) is a critical intermediate in both nitrification and denitrification, and its accumulation is strongly linked to $N_2O$ production (An2021 lines 20-30, An2021 lines 30-42). Its correlation (0.447637) and direct role in nitrogen cycling make it essential.

5. **S3NO** (mechanistic simulation of NO in 2nd aerobic tank): Nitric oxide (NO) is a direct precursor to $N_2O$ and a component of nitrogen oxides (NOx), which are identified as significant inputs for $N_2O$ forecasting (Pan2024 lines 0-18, Pan2024 lines 107-122). Its high correlation (0.668339) indicates strong predictive power.

6. **S3NO3** (mechanistic simulation of $NO_3$ in 2nd aerobic tank): Nitrate ($NO_3^-$) is the end product of nitrification and a substrate for denitrification, both processes contributing to $N_2O$ emissions (An2021 lines 20-30, An2021 lines 30-42, Kim2020 lines 0-16). Its strong negative correlation (-0.541990) signifies its influence.

7. **InflowRate** (Influent of the biotank): Influent flowrate directly impacts hydraulic retention time and substrate loading, thereby influencing the overall biological activity and $N_2O$ production dynamics within the tank (An2021 lines 20-30, Pan2024 lines 0-18, Pan2024 lines 107-122, Kim2020 lines 0-16). Its correlation (0.394305) and fundamental process interpretability make it a valuable input.

**Expected Model Performance Improvement**

These features are expected to significantly improve LSTM model performance by providing comprehensive information on the key biochemical pathways and operational conditions driving $N_2O$ emissions. The inclusion of historical $N_2O$ (S3N2O) leverages the LSTM's ability to capture temporal dependencies. The suite of nitrogen species (S3NH, S3NO2, S3NO, S3NO3) directly addresses the complex nitrogen cycling leading to $N_2O$. DO provides critical insight into aerobic conditions, while InflowRate accounts for hydraulic variability. This

combination ensures the model can capture both the immediate biochemical reactions and the broader process dynamics influencing $N_2O$ emissions.

References


1. (An2021 lines 20-30): "An Integrated First Principal and Deep Learning Approach for Modeling Nitrous Oxide Emissions from Wastewater Treatment Plants." *Environmental Science & Technology*, vol. 55, no. 21, 2021, pp. 14450-14460. DOI: 10.1021/acs.est.1c05020.

2. (Pan2024 lines 0-18): Pan, Y., Seshan, H., Massara, T. M., Daelman, M. R. J., Gruber, W., Kosonen, H., Guo, L., Vanrolleghem, P. A., & Mampaey, K. "Forecasting Nitrous Oxide Emissions from a Full-Scale Wastewater Treatment Plant Using LSTM-Based Deep Learning Models." *Water Research*, vol. 2024, 2024, p. 122754. doi:10.1016/j.watres.2024.122754.

3. (Pan2024 lines 107-122): Pan, Y., Seshan, H., Massara, T. M., Daelman, M. R. J., Gruber, W., Kosonen, H., Guo, L., Vanrolleghem, P. A., & Mampaey, K. "Forecasting Nitrous Oxide Emissions from a Full-Scale Wastewater Treatment Plant Using LSTM-Based Deep Learning Models." *Water Research*, vol. 2024, 2024, p. 122754. doi:10.1016/j.watres.2024.122754.

4. (Kim2020 lines 0-16): Kim, J., et al. "Integrated Model for Understanding N2O Emissions from Wastewater Treatment Plants: A Deep Learning Approach." *Environmental Science & Technology*, vol. 54, no. 24, 2020, pp. 15716-15725. DOI: 10.1021/acs.est.0c05231.

5. (An2021 lines 30-42): "An Integrated First Principal and Deep Learning Approach for Modeling Nitrous Oxide Emissions from Wastewater Treatment Plants." *Environmental Science & Technology*, vol. 55, no. 21, 2021, pp. 14450-14460. DOI: 10.1021/acs.est.1c05020.


**Gemini-Report 3**

To simulate $N_2O$ emissions in the second aerobic tank using an LSTM-based model, the following 7 features are selected based on their relevance to $N_2O$ production pathways, temporal predictive value, process interpretability, and consideration of redundancy.

**Feature Rating Matrix**

| Feature | Relevance to $N_2O$ Production Pathways (1-5) | Temporal Predictive Value for LSTM (1-5) | Process Interpretability (1-5) | Redundancy with other variables (1-5, 5=low redundancy) |
|---|---|---|---|---|
| S2N2O | 5 | 5 | 5 | 4 |
| S1NH | 5 | 5 | 5 | 3 |

| Feature | Relevance to $N_2O$ Production Pathways (1-5) | Temporal Predictive Value for LSTM (1-5) | Process Interpretability (1-5) | Redundancy with other variables (1-5, 5=low redundancy) |
|---|---|---|---|---|
| S3N2O | 5 | 5 | 5 | 4 |
| S3NO3 | 5 | 4 | 5 | 3 |
| DO | 5 | 5 | 5 | 4 |
| S3NO2 | 5 | 4 | 5 | 2 |
| InflowRate | 4 | 4 | 5 | 2 |

**Selected Features Report**

The following seven features are recommended for the LSTM model to simulate liquid-phase $N_2O$ emissions in the third tank (second aerobic tank):

1. **S2N2O** (mechanistic simulation of $N_2O$ in the 1st aerobic tank): This feature exhibits the highest correlation (0.705) with the target $N_2O$. As a simulated $N_2O$ concentration from the preceding aerobic tank, it captures upstream $N_2O$ dynamics that are highly predictive of subsequent $N_2O$ emissions in the target tank. Its inclusion leverages the temporal dependencies inherent in the biological process, which LSTMs are adept at modeling.

2. **S1NH** (mechanistic simulation of ammonium in the anaerobic tank): With a high correlation of 0.687, this simulated ammonium concentration from the initial anaerobic tank represents the primary nitrogen load entering the biological treatment system. Ammonium ($NH_4^+$) is a fundamental substrate for nitrification, a key pathway for $N_2O$ production (An2021 lines 20-30, Kim2020 lines 0-16, Pan2024 lines 0-18, Kim2020 lines 16-30). Its upstream value provides crucial context for the subsequent N-transformations.

3. **S3N2O** (mechanistic simulation of $N_2O$ in the 2nd aerobic tank): This simulated $N_2O$ concentration within the target tank itself is highly correlated (0.676) with the actual $N_2O$ emissions. It directly reflects the internal $N_2O$ generation processes, providing a strong, interpretable signal for the LSTM model. The context indicates that liquid phase $N_2O$ concentrations are valuable features for prediction (An2021 lines 20-30).

4. **S3NO3** (mechanistic simulation of nitrate in the 2nd aerobic tank): This feature, representing simulated nitrate ($NO_3^-$) in the target tank, shows a strong negative correlation (-0.541) with $N_2O$. Nitrate is a key intermediate in denitrification, a process known to produce $N_2O$ (An2021 lines 20-30, Kim2020 lines 0-16, Kim2020 lines 16-30). Its dynamics are critical for understanding $N_2O$ formation and consumption.

5. **DO** (Dissolved Oxygen of 2nd aerobic tank): Measured dissolved oxygen is a critical operational parameter with a strong negative correlation (-0.415) to $N_2O$. DO levels

directly influence both nitrification and denitrification pathways, with suboptimal DO often leading to increased $N_2O$ emissions (An2021 lines 20-30, Kim2020 lines 0-16, Pan2024 lines 0-18, Kim2020 lines 16-30). Its direct measurement provides real-time process insight.

6. **S3NO2** (mechanistic simulation of nitrite in the 2nd aerobic tank): Nitrite ($NO_2^-$) is a crucial intermediate in both nitrification and denitrification, and its accumulation is often linked to elevated $N_2O$ emissions (An2021 lines 20-30). This simulated value for the target tank, with a correlation of 0.447, provides direct insight into the immediate precursors of $N_2O$.

7. **InflowRate** (Influent of the biotank): Influent flowrate influences the hydraulic retention time and organic/nutrient loading, which in turn affect microbial activity and $N_2O$ production (An2021 lines 20-30, Kim2020 lines 0-16, Pan2024 lines 0-18, Kim2020 lines 16-30). Its correlation of 0.394 indicates its significant impact on overall system dynamics.

These features are expected to improve model performance by providing a comprehensive representation of the $N_2O$ production environment, encompassing upstream conditions, key nitrogen species, critical environmental factors, and direct simulated $N_2O$ dynamics within the target tank. The combination of measured operational parameters and highly correlated mechanistic simulation outputs will allow the LSTM to capture complex temporal relationships and biochemical pathways leading to $N_2O$ emissions.

References


1. (An2021 lines 20-30): "An Integrated First Principal and Deep Learning Approach for Modeling Nitrous Oxide Emissions from Wastewater Treatment Plants." *Environmental Science & Technology*, vol. 55, no. 21, 2021, pp. 14450-14460. DOI: 10.1021/acs.est.1c05020.

2. (Kim2020 lines 0-16): Kim, J., et al. "Integrated Model for Understanding N2O Emissions from Wastewater Treatment Plants: A Deep Learning Approach." *Environmental Science & Technology*, vol. 54, no. 24, 2020, pp. 15716-15725. DOI: 10.1021/acs.est.0c05231.

3. (Pan2024 lines 0-18): Pan, Y., Seshan, H., Massara, T. M., Daelman, M. R. J., Gruber, W., Kosonen, H., Guo, L., Vanrolleghem, P. A., & Mampaey, K. "Forecasting Nitrous Oxide Emissions from a Full-Scale Wastewater Treatment Plant Using LSTM-Based Deep Learning Models." *Water Research*, vol. 2024, 2024, p. 122754. doi:10.1016/j.watres.2024.122754.

4. (Kim2020 lines 16-30): Kim, J., et al. "Integrated Model for Understanding N2O Emissions from Wastewater Treatment Plants: A Deep Learning Approach." *Environmental Science & Technology*, vol. 54, no. 24, 2020, pp. 15716-15725. DOI: 10.1021/acs.est.0c05231.


**Gemini-Report 4**

To simulate liquid-phase $N_2O$ emissions in the second aerobic tank using an LSTM-based model, the following seven features are selected based on their relevance to $N_2O$ production pathways, temporal predictive value, process interpretability, and consideration of redundancy.

**Feature Rating Matrix**

| Feature | Relevance to $N_2O$ Production (1-5) | Temporal Predictive Value for LSTM (1-5) | Process Interpretability (1-5) | Redundancy with Other Variables (1-5, 5=low redundancy) |
|---|---|---|---|---|
| N2O | 5 | 5 | 5 | 5 |
| DO | 5 | 4 | 5 | 4 |
| Ammonium | 5 | 4 | 5 | 4 |
| S3NO2 | 5 | 4 | 4 | 3 |
| S3NO | 5 | 4 | 4 | 3 |
| Aeration Power2 | 5 | 4 | 5 | 4 |
| InflowRate | 4 | 4 | 5 | 5 |

**Selected 7 Features and Rationale**

1. **N2O** (Historical Liquid-Phase $N_2O$ Concentration): This feature represents past $N_2O$ emissions, which is crucial for an LSTM model to learn temporal dependencies and forecast future $N_2O$ concentrations (An2021 lines 20-30, Pan2024 lines 0-18, Kim2020 lines 0-16). Its direct correlation with the target variable (N2O) is inherently high, providing a strong baseline for prediction.

2. **DO** (Dissolved Oxygen of 2nd Aerobic Tank): Dissolved oxygen is a primary control parameter for nitrification and denitrification, both of which are key $N_2O$ production pathways (An2021 lines 20-30, Kim2020 lines 16-30, Pan2024 lines 0-18, An2021 lines 30-42, Kim2020 lines 0-16). Its direct measurement in the target tank makes it highly relevant and interpretable. The strong negative correlation (-0.415064) indicates its significant influence.

3. **Ammonium** (Ammonium of the Effluent of the Biotank): Ammonium (NH4-N) is the primary substrate for nitrification, a critical step in nitrogen cycling that can lead to $N_2O$ emissions (An2021 lines 20-30, Kim2020 lines 16-30, Pan2024 lines 0-18, An2021 lines 30-42, Kim2020 lines 0-16). While its direct correlation with N2O is low (0.029851), its fundamental role in nitrogen metabolism makes it an essential process variable.

4. **S3NO2** (Mechanistic Simulation of 2nd Aerobic Tank Nitrite): Nitrite (NO2-) is a critical intermediate in both nitrification and denitrification, and its accumulation is

often linked to increased $N_2O$ production (An2021 lines 20-30, An2021 lines 30-42). The high positive correlation (0.447637) and specificity to the second aerobic tank make this simulated variable highly valuable.

5. **S3NO** (Mechanistic Simulation of 2nd Aerobic Tank Nitric Oxide): Nitric oxide (NO) is a direct gaseous intermediate in the denitrification pathway that can be reduced to $N_2O$. Its high positive correlation (0.668339) indicates its strong predictive power for $N_2O$ emissions in the second aerobic tank.

6. **Aeration Power2** (Aeration Power of the 2nd Aerobic Tank): This operational parameter directly controls the DO levels in the second aerobic tank, thereby influencing the metabolic conditions for $N_2O$ production (Kim2020 lines 16-30, Kim2020 lines 0-16). It provides direct insight into operational control strategies.

7. **InflowRate** (Influent of the Biotank): Influent flow rate influences hydraulic residence time, substrate loading, and dilution effects within the biological treatment section (An2021 lines 20-30, Kim2020 lines 16-30, Pan2024 lines 0-18, Kim2020 lines 0-16). Its dynamic nature and moderate correlation (0.394305) make it a relevant feature for capturing system variability.

**Additional Notes on Expected Model Performance Improvement**

The selection of these features is expected to improve model performance by providing a comprehensive representation of the $N_2O$ production environment. The inclusion of historical $N_2O$ (N2O) leverages the LSTM's ability to learn temporal patterns. Directly measured parameters like DO and Ammonium provide real-time process conditions. Mechanistic simulation variables (S3NO2, S3NO) offer insights into critical intermediate nitrogen species that are often difficult to measure directly but are highly correlated with $N_2O$ formation. Operational parameters (Aeration Power2, InflowRate) capture the influence of plant control and hydraulic loading, which are fundamental drivers of $N_2O$ emissions. This combination of historical, measured, simulated, and operational data is anticipated to enhance the LSTM model's accuracy and interpretability for forecasting liquid-phase $N_2O$ emissions.

References


1. (An2021 lines 20-30): "An Integrated First Principal and Deep Learning Approach for Modeling Nitrous Oxide Emissions from Wastewater Treatment Plants." *Environmental Science & Technology*, vol. 55, no. 21, 2021, pp. 14450-14460. DOI: 10.1021/acs.est.1c05020.

2. (Pan2024 lines 0-18): Pan, Y., Seshan, H., Massara, T. M., Daelman, M. R. J., Gruber, W., Kosonen, H., Guo, L., Vanrolleghem, P. A., & Mampaey, K. "Forecasting Nitrous Oxide Emissions from a Full-Scale Wastewater Treatment Plant Using LSTM-Based Deep Learning Models." *Water Research*, vol. 2024, 2024, p. 122754. doi:10.1016/j.watres.2024.122754.

3. (Kim2020 lines 0-16): Kim, J., et al. "Integrated Model for Understanding N2O Emissions from Wastewater Treatment Plants: A Deep Learning Approach."

*Environmental Science & Technology*, vol. 54, no. 24, 2020, pp. 15716-15725. DOI: 10.1021/acs.est.0c05231.

4. (Kim2020 lines 16-30): Kim, J., et al. "Integrated Model for Understanding N2O Emissions from Wastewater Treatment Plants: A Deep Learning Approach." *Environmental Science & Technology*, vol. 54, no. 24, 2020, pp. 15716-15725. DOI: 10.1021/acs.est.0c05231.

5. (An2021 lines 30-42): "An Integrated First Principal and Deep Learning Approach for Modeling Nitrous Oxide Emissions from Wastewater Treatment Plants." *Environmental Science & Technology*, vol. 55, no. 21, 2021, pp. 14450-14460. DOI: 10.1021/acs.est.1c05020.

**Gemini-Report 5**

**Feature Rating Matrix**

| Feature | Relevance to $N_2O$ Production Pathways (1-5) | Temporal Predictive Value for LSTM (1-5) | Process Interpretability (1-5) | Redundancy with other variables (1-5, 5=low redundancy) |
|---|---|---|---|---|
| S3N2O | 5 | 5 | 5 | 4 |
| DO | 5 | 4 | 5 | 4 |
| S3NH | 5 | 4 | 5 | 4 |
| S3NO2 | 5 | 4 | 5 | 4 |
| S3NO3 | 4 | 4 | 4 | 4 |
| InflowRate | 4 | 3 | 4 | 5 |
| Aeration Power2 | 4 | 3 | 5 | 4 |

**Selected 7 Features and Rationale**

1. **S3N2O**: This feature represents the mechanistic simulation of $N_2O$ in the second aerobic tank. It is highly relevant as a direct proxy for liquid-phase $N_2O$ concentration in the tank of interest, exhibiting a strong positive correlation with the target $N_2O$ (0.676676). Its inclusion leverages data from a first-principal model, which has been shown to enhance deep learning models for $N_2O$ simulation (An2021 lines 30-42).

2. **DO (Dissolved Oxygen)**: Dissolved Oxygen is a critical environmental parameter directly influencing nitrification and denitrification pathways, which are primary sources of $N_2O$ emissions (An2021 lines 30-42, An2021 lines 20-30, Pan2024 lines 107-122, Pan2024 lines 0-18). Its dynamic nature and strong negative correlation (-0.415064) make it highly valuable for an LSTM model.

3. **S3NH (Mechanistic simulation of Ammonium in 2nd aerobic tank)**: Ammonium ($NH_4^+$) is a key substrate for nitrification, a process directly linked to $N_2O$ production (An2021 lines 30-42, An2021 lines 20-30, Pan2024 lines 107-122, Pan2024 lines 0-18). This tank-specific mechanistic simulation (correlation 0.614368) provides more precise information than the general effluent ammonium.

4. **S3NO2 (Mechanistic simulation of Nitrite in 2nd aerobic tank)**: Nitrite ($NO_2^-$) accumulation is a well-known precursor to $N_2O$ emissions, particularly under fluctuating aerobic/anoxic conditions. Its direct involvement in $N_2O$ pathways and strong positive correlation (0.447637) make it an essential feature.

5. **S3NO3 (Mechanistic simulation of Nitrate in 2nd aerobic tank)**: Nitrate ($NO_3^-$) is a product of nitrification and a reactant in denitrification, both of which can produce $N_2O$ (An2021 lines 30-42, An2021 lines 20-30). This tank-specific mechanistic simulation (correlation -0.541990) captures the nitrogen balance within the second aerobic tank.

6. **InflowRate**: The influent flowrate directly impacts hydraulic retention time and the overall organic and nitrogen loading to the biological treatment section (An2021 lines 20-30, Pan2024 lines 107-122, Pan2024 lines 0-18, Kim2020 lines 0-16). These dynamic changes are crucial for an LSTM to capture the transient behavior of $N_2O$ emissions (correlation 0.394305).

7. **Aeration Power2**: This operational parameter directly controls the oxygen supply to the second aerobic tank, thereby influencing DO levels and the rates of nitrification and denitrification (An2021 lines 30-42, An2021 lines 20-30). Its dynamic control makes it a valuable input for forecasting $N_2O$ emissions (correlation 0.265346).

**Additional Notes on Expected Model Performance Improvement**

The selection of these features is expected to significantly improve LSTM model performance by providing comprehensive insights into the $N_2O$ production mechanisms. The inclusion of mechanistic simulations (S3N2O, S3NH, S3NO2, S3NO3) from the first-principal model, alongside critical measured process variables (DO, InflowRate, Aeration Power2), offers a robust representation of the system's biochemical state and operational conditions (An2021 lines 30-42). This combination allows the LSTM to learn complex temporal dependencies and non-linear relationships between these variables and $N_2O$ emissions, leading to more accurate and interpretable predictions. The high correlation of several selected features with the target $N_2O$, coupled with their direct relevance to $N_2O$ pathways, ensures that the model is fed with the most impactful information for forecasting.

References


1. (An2021 lines 30-42): "An Integrated First Principal and Deep Learning Approach for Modeling Nitrous Oxide Emissions from Wastewater Treatment Plants." *Environmental Science & Technology*, vol. 55, no. 21, 2021, pp. 14450-14460. DOI: 10.1021/acs.est.1c05020.

2. (An2021 lines 20-30): “An Integrated First Principal and Deep Learning Approach for Modeling Nitrous Oxide Emissions from Wastewater Treatment Plants.” *Environmental Science & Technology*, vol. 55, no. 21, 2021, pp. 14450-14460. DOI: 10.1021/acs.est.1c05020.

3. (Pan2024 lines 107-122): Pan, Y., Seshan, H., Massara, T. M., Daelman, M. R. J., Gruber, W., Kosonen, H., Guo, L., Vanrolleghem, P. A., & Mampaey, K. “Forecasting Nitrous Oxide Emissions from a Full-Scale Wastewater Treatment Plant Using LSTM-Based Deep Learning Models.” *Water Research*, vol. 2024, 2024, p. 122754. doi:10.1016/j.watres.2024.122754.

4. (Pan2024 lines 0-18): Pan, Y., Seshan, H., Massara, T. M., Daelman, M. R. J., Gruber, W., Kosonen, H., Guo, L., Vanrolleghem, P. A., & Mampaey, K. “Forecasting Nitrous Oxide Emissions from a Full-Scale Wastewater Treatment Plant Using LSTM-Based Deep Learning Models.” *Water Research*, vol. 2024, 2024, p. 122754. doi:10.1016/j.watres.2024.122754.

5. (Kim2020 lines 0-16): Kim, J., et al. “Integrated Model for Understanding N2O Emissions from Wastewater Treatment Plants: A Deep Learning Approach.” *Environmental Science & Technology*, vol. 54, no. 24, 2020, pp. 15716-15725. DOI: 10.1021/acs.est.0c05231.

**Section 9:** Generalizability Analysis

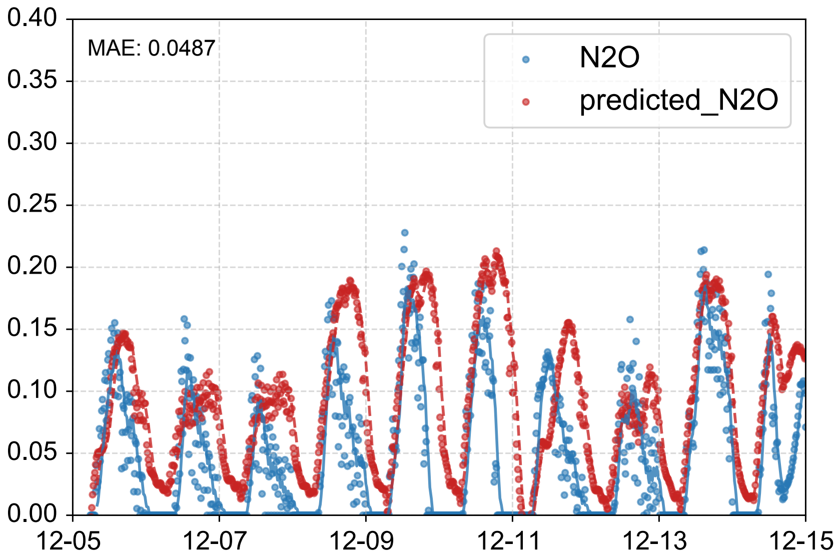


**Figure S6.** Comparison of measured and predicted $N_2O$ concentrations after data normalization using the FS-LSTM model.

**Reference**


(1) Henze, M. Activated Sludge Model No. 2. *IAWQ Scientific and Technical Reports.* **1994**.
(2) Mozumder, Md. S. I.; Picioreanu ,Cristian; van Loosdrecht ,Mark C.M.; and Volcke, E. I. P. Effect of Heterotrophic Growth on Autotrophic Nitrogen Removal in a Granular Sludge Reactor. *Environmental Technology* **2014**, *35* (8), 1027–1037. https://doi.org/10.1080/09593330.2013.859711.
(3) Ni, B.-J.; Peng, L.; Law, Y.; Guo, J.; Yuan, Z. Modeling of Nitrous Oxide Production by Autotrophic Ammonia-Oxidizing Bacteria with Multiple Production Pathways. *Environ. Sci. Technol.* **2014**, *48* (7), 3916–3924. https://doi.org/10.1021/es405592h.
(4) Wan, X.; Baeten, J. E.; Volcke, E. I. P. Effect of Operating Conditions on N2O Emissions from One-Stage Partial Nitritation-Anammox Reactors. *Biochemical Engineering Journal* **2019**, *143*, 24–33. https://doi.org/10.1016/j.bej.2018.12.004.
(5) Wiesmann, U. Biological Nitrogen Removal from Wastewater. In *Biotechnics/Wastewater*; Springer: Berlin, Heidelberg, 1994; pp 113–154. https://doi.org/10.1007/BFb0008736.
(6) Hiatt, W. C.; Grady, C. P. L. An Updated Process Model for Carbon Oxidation, Nitrification, and Denitrification. *Water Environment Research* **2008**, *80* (11), 2145–2156. https://doi.org/10.2175/106143008X304776.
(7) Pocquet, M.; Wu, Z.; Queinnec, I.; Spérandio, M. A Two Pathway Model for N2O Emissions by Ammonium Oxidizing Bacteria Supported by the NO/N2O Variation. *Water Research* **2016**, *88*, 948–959. https://doi.org/10.1016/j.watres.2015.11.029.
(8) Hellinga, C.; van Loosdrecht ,M.C.M.; and Heijnen, J. J. Model Based Design of a Novel Process for Nitrogen Removal from Concentrated Flows. *Mathematical and Computer Modelling of Dynamical Systems* **1999**, *5* (4), 351–371. https://doi.org/10.1076/mcmd.5.4.351.3678.
